# Horizontal gene transfer of zinc and non-zinc forms of bacterial ribosomal protein S4

**DOI:** 10.1186/1471-2148-9-179

**Published:** 2009-07-29

**Authors:** Ke Chen, Elijah Roberts, Zaida Luthey-Schulten

**Affiliations:** 1 Center for Biophysics and Computational Biology, University of Illinois at Urbana-Champaign, Urbana, IL 61801, USA; 2 Department of Chemistry, University of Illinois at Urbana-Champaign, Urbana, IL 61801, USA; 3 Institute for Genomic Biology, University of Illinois at Urbana-Champaign, Urbana, IL 61801, USA

## Abstract

**Background:**

The universal ribosomal protein S4 is essential for the initiation of small subunit ribosomal assembly and translational accuracy. Being part of the information processing machinery of the cell, the gene for S4 is generally thought of as being inherited vertically and has been used in concatenated gene phylogenies. Here we report the evolution of ribosomal protein S4 in relation to a broad sharing of zinc/non-zinc forms of the gene and study the scope of horizontal gene transfer (HGT) of S4 during bacterial evolution.

**Results:**

In this study we present the complex evolutionary history of ribosomal protein S4 using 660 bacterial genomes from 16 major bacterial phyla. According to conserved characteristics in the sequences, S4 can be classified into C+ (zinc-binding) and C- (zinc-free) variants, with 26 genomes (mainly from the class *Clostridia*) containing genes for both. A maximum likelihood phylogenetic tree of the S4 sequences was incongruent with the standard bacterial phylogeny, indicating a departure from strict vertical inheritance. Further analysis using the genome content near the S4 genes, which are usually located in a conserved gene cluster, showed not only that HGT of the C- gene had occurred at various stages of bacterial evolution, but also that both the C- and C+ genes were present before the individual phyla diverged. To explain the latter, we theorize that a gene pool existed early in bacterial evolution from which bacteria could sample S4 gene variants, according to environmental conditions. The distribution of the C+/- variants for seven other zinc-binding ribosomal proteins in these 660 bacterial genomes is consistent with that seen for S4 and may shed light on the evolutionary pressures involved.

**Conclusion:**

The complex history presented for "core" protein S4 suggests the existence of a gene pool before the emergence of bacterial lineages and reflects the pervasive nature of HGT in subsequent bacterial evolution. This has implications for both theoretical models of evolution and practical applications of phylogenetic reconstruction as well as the control of zinc economy in bacterial cells.

## Background

The ribosome is an elaborate ribonucleoprotein complex whose evolution is intrinsically linked with that of the cell. It has been recognized since the 1970's that the molecular core of the ribosome was in place before the divergence of the three primary organismal lineages, *Bacteria*, *Archaea*, and *Eucarya *(the domains of life). The history of these lineages, as inferred from the ribosomal RNA (rRNA) and represented by the universal phylogenetic tree (UPT) [1], provides an organismal reference by which the evolutionary history of a gene can be studied. Despite conservation of a large portion of the ribosomal structure among the lineages, the ribosomes of each domain of life contain certain sequence and structural signatures that are unique to and constant within the domain. Such signatures have been identified in both the rRNA and ribosomal proteins (r-proteins), including many r-proteins that are specific to one of the primary lineages. This suggests that both large and small scale changes in the ribosome were still evolving after the domains diverged and then spreading among all of a domain's developing sub-branches [2]. The exact mechanism by which homogenization of the branches might have occurred is unclear and certainly a matter of some debate, but pervasive horizontal gene transfer (HGT) among aboriginal cellular life [3,4] is one possible mechanism. Studying the pattern by which such a signature spread among the evolving lineages can help resolve the dynamics of the evolutionary process at the time.

Horizontal gene transfer, the acquisition of non-inherited genetic material, is widely regarded as a common and important evolutionary phenomenon [5-10]. It is now understood that HGT allows microorganisms to break out of strictly clonal, bifurcating lineages in their search for genetic innovation [11]. Despite the complexity of the ribosome and the potential for malfunction from acquiring a new version of a single ribosomal component, r-protein genes are known to have been horizontally transferred within a domain of life, although no inter-domain HGT has been identified. The first instance of HGT of an r-protein gene was reported by Brochier *et al*. [12] for S14. In their study, they classified the bacterial S14 sequences into distinct groups based on characteristic insertions/deletions (indels) and presented phylogenetic evidence that, in some cases, the groups were at odds with the classical bacterial phylogeny. They argued that these discrepancies, as well as unusual gene ordering and duplications in the affected lineages, were the result of ancient HGT events. Thus, they proposed that there must have been some evolutionary pressure favoring the fixation of the transferred r-protein gene, in accordance with the "complexity hypothesis" of Jain *et al*. [13] regarding the lower probability of HGT for informational genes.

A later bioinformatics study further extended analysis of HGT and gene duplication in the r-proteins. Using genomes of thirty bacteria and genomic data for r-proteins of mitochondria and chloroplasts from seven eukaryotic organisms, Makarova *et al*. [14] found six additional ribosomal proteins (S18, L28, L31, L32, L33, L36) that shared similar evolutionary patterns to S14 within the bacterial lineage, including discrepancies in genome organization and gene copy number. Furthermore, they showed that the phylogenetic patterns were related to the zinc binding ability of the r-proteins. Two variants were found of each r-protein: one containing a zinc finger motif with four conserved cysteine residues (or occasionally three cysteine and one histidine residue) and another with a complete or partial disruption of the motif. The two variants were referred to as C+ and C-, respectively. Their data suggested that in each case the C+ variant was the ancestral form and that ancient gene duplication followed by disruption of the zinc finger in the paralog and later loss of the original C+ gene in some lineages (differential gene loss; DGL) was the major evolutionary pattern with HGT also occasionally occurring.

Following initial identification of C- variants of bacterial zinc-binding r-proteins, other laboratories began investigating their regulation in organisms with both C+ and C- genes to better understand the evolutionary pressures giving rise to the C- forms. It was predicted theoretically [15] and then found experimentally, first in *Bacillus subtilis *for S14 and L31 [16,17] and then in *Streptomyces coelicolor *for S14, L28, L31, L32, L33, and L36 [18] and in *Mycobacterium tuberculosis *for S14, S18, L28, and L33 [19], that the paralogous C- versions of some r-protein genes were up-regulated under conditions of low zinc. These groups proposed that the C- paralogs served two possible functions, release of free zinc into the cell in low zinc environments (by ribosomal exchange with the endogenous C+ protein) and/or continued production of ribosomes under zinc-limiting conditions. Whether these are the only pressures that gave rise to the C- forms is unknown, but it is clear that some ribosomal proteins have a unique and interesting evolutionary history related to zinc binding.

All seven zinc binding r-proteins discussed above, except for S14, are unique to *Bacteria*. Such domain specific r-proteins are signatures of the bacterial ribosome. Roberts *et al*. [2] showed that the signatures are not limited to just entire domain specific ribosomal proteins, but can also take the form of domain specific insertions in the r-proteins that are universally distributed among all three domains of life. Such a case is found in the universal r-protein S4, a two domain protein ~200 amino acids in length that is essential for the initiation of small subunit (SSU) ribosomal assembly and translational accuracy. The C-terminal domain of S4 (residues 46–206; all residues given in terms of *Escherichia coli *numbering) is known to be an RNA binding domain, binding to both rRNA and messenger RNA (mRNA) [20-22], and is homologous between *Bacteria *and *Archaea*. While the overall sequence identity for the C-terminal domain is only 36% among bacteria and 32% across all domains of life, the region making contact with the ribosomal RNA is conserved with an average sequence identity of 46% and 40% respectively. The N-terminal domain, in contrast, appears to be non-homologous between *Bacteria *and *Archaea *and was identified in [2] as a bacterial-specific structural signature that coevolved with a bacterial specific extension of an RNA helix (helix h16) on the 16S rRNA. Alone, the bacterial S4 structure has been determined only without the unstructured N-terminus [23], but when complexed with the ribosome the structure of the full protein has been determined. The crystal structure of the *Thermus thermophilus *ribosome [24] shows that the N-terminal domain of S4 contains a zinc finger motif ligated to a zinc atom and the sequence analysis presented here shows conservation of the four cysteine residues in the zinc-finger motif only in a subset of the bacterial lineages. This variation in zinc binding ability within the bacterial lineages of S4 was overlooked in previous studies of the evolutionary history of zinc-binding r-proteins.

The recent growth in the number of available bacterial genome sequences allows a broad evolutionary history of a gene to be reconstructed, especially in regard to HGT [25,26]. Besides sequence data for phylogenetic reconstructions, full genomes provide data on genome organization and gene distribution, which are particularly useful in aiding interpretation of possible HGT events. In this study, we use 660 available bacterial genomes to study the evolution of ribosomal protein S4 in the bacteria. We find that S4 can be classified into C+ and C- variants (zinc binding and non-zinc binding, respectively), with multiple independent origins of the C- form. A maximum likelihood tree of S4 shows disagreement with the standard bacterial phylogeny, indicating a more complex evolutionary history than previously known. Considering the fact that the S4 gene is part of a highly conserved gene cluster in bacteria consisting of the *S10-spc-α *operons [27], we see surprising evidence for the endogenous origin of the C- form in some phyla and hypothesize that both the C+ and C- forms may have been present before the bacterial phyla diverged with different lineages sampling from the variants according to the local environment. In accordance with this hypothesis, we also present evidence that C- paralogous copies in genomes containing both variants of S4, as well as all S4 genes outside the *α*-operon, are results of HGT events. Regulation of the paralogous S4 genes seems to differ from the zinc-binding r-proteins previously identified, and the expanded distribution of the C+/C- variants in all the zinc-binding r-proteins we present may provide insight on the evolution of zinc usage in bacterial lineages.

## Results

### Sequence alignment and classification of bacterial r-protein S4

**Figure 1:**
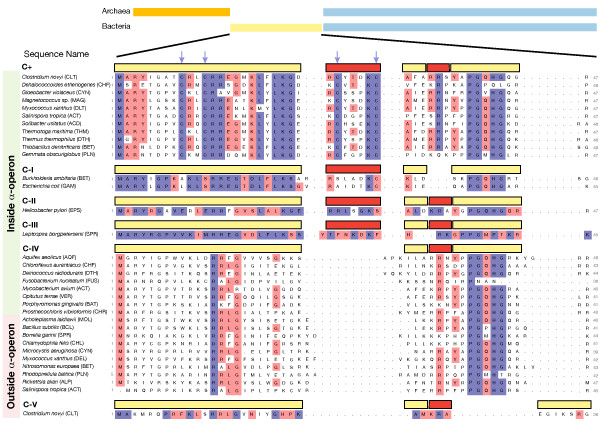
**Multiple sequence alignment of ribosomal protein S4**. Shown is a representative sample of the full sequence alignment. Sequences are grouped according to specific sequence characteristics (see text) and positions are colored by conservation within the group at (blue) 95% and (red) 70%. The arrows above the alignment indicate positions of the two pairs of cysteine residues. The three-letter abbreviations indicate the phylum or class that the organisms belong to: ACD (*Acidobacteria*), ACT (*Actinobacteria*), ALP (*Alphaproteobacteria*), AQF (*Aquificae*), BAT (*Bacteroidetes*), BET (*Betaproteobacteria*), CHF (*Chloroflexi*), CHL (*Chlamydiae*), CHR (*Chlorobi*), CLT (*Clostridia*), CYN (*Cyanobacteria*), DEL (*Deltaproteobacteria*), DTH (*Deinococcus-Thermus*), EPS (*Epsilonproteobacteria*), FUS (*Fusobacteria*), GAM (*Gammaproteobacteria*), MAG (*Magnetococcus*), MOL (*Mollicutes*), PLN (*Planctomycetes*), SPR (*Spirochaetes*), VER (*Verrucomicrobia*).

To study the history of S4 in bacteria, we first extracted 688 sequences of S4 and paralogs from 660 complete and draft bacterial genomes (complete list in Additional File 1). We then constructed a multiple sequence alignment (MSA) of the sequences using automated alignment tools followed by manual correction (see Methods). Any evolutionary study of a large set of diverse organisms is likely to reveal a complex history, so to aid further analysis of the relationships between the sequences we classified them into six types using key sequence and structural signatures that define apparently monophyletic groups (such features are also known as synapomorphies). These types classify the sequences according to the presence of or disruption pattern in the zinc finger motif. We find one C+ version (with four conserved cysteines) and five C- subtypes (with various patterns of loss of the zinc finger). C-(I), C-(II) and C-(III) sequences possess a seven residue indel present in the C+ type but show gradual loss of the four cysteines (from two to one to zero). C-(IV) and C-(V) subtypes are missing the indel characteristic of the C+ type as well as all four cysteines. Further distinctions between the C- subtypes are based on sequence signatures in the N-terminus. Figure 1 shows a sample of the N-terminal portion of the alignment from all of the major bacterial phyla grouped according to these classifications.

From a conservation analysis of the MSA, it is apparent that the S4 sequences can be broadly classified into C+ and C- variants (following the notation introduced by Makarova *et al*. [14]) based on the conservation of four cysteine residues in the N-terminal domain. C+ type sequences contain two conserved pairs of cysteine residues in a "CXXC...CXXXXC" motif. The first pair appears near the beginning of the sequence (at residues 9 and 12) and the second pair in a seven residue segment that is an insertion relative to most of the C- sequences (the first red block in Figure 1). As shown in the *T. thermophilus *ribosome structure, these four cysteine residues bind a zinc ion. Within the C+ group, the N-terminal domain is highly conserved with an average percent sequence identity of 65%. The C+ group includes sequences from diverse bacteria groups: *Acidobacteria*, *Actinobacteria*, *Chloroflexi*, *Clostridia*, *Cyanobacteria*, *Deinococcus-Thermus*, *Planctomycetes*, *Proteobacteria *(*Beta *and *Delta *classes) and *Thermotogae*.

The C- variants of S4 show less homogeneity than their C+ counterparts. By definition, they all lack the four cysteine residues, but other characteristic features in the N-terminus allow them to be further classified according to their likely evolutionary origin. The major distinguishing feature between the C-forms is the presence or absence of the seven residue indel that contains the second pair of cysteine residues in the C+ form. Three C- types, C-(I), C-(II), and C-(III), possess the indel, but have disruption of the zinc binding motif. Each of these types is confined to a small portion of the bacterial tree, while sequences containing the indel with the conserved cysteine residues are seen in a wide variety of bacteria. This difference suggests that each of these three groups may have been formed by relatively recent, independent mutations of an ancestral C+ form.

**Figure 2:**
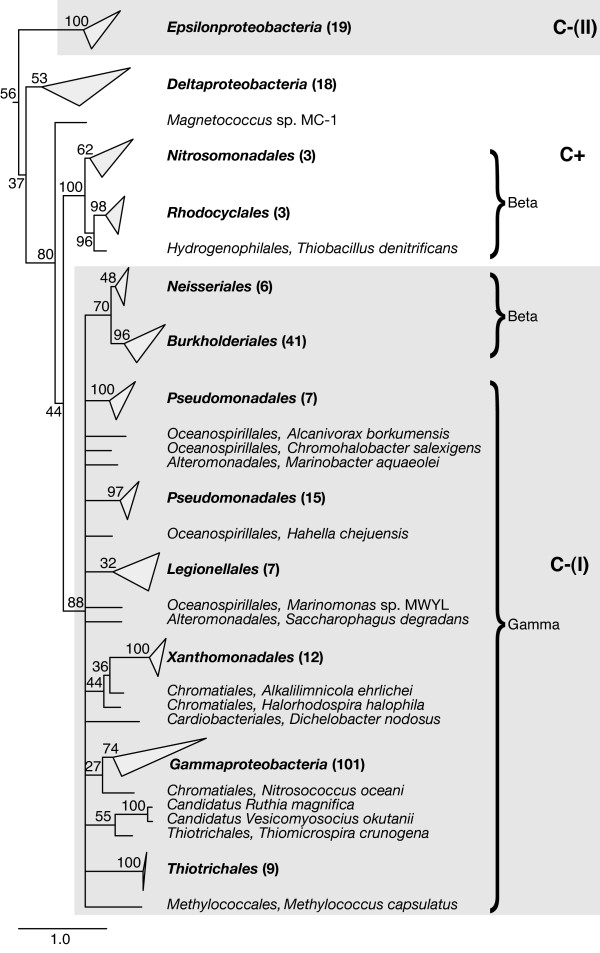
**Consensus phylogenetic tree of ribosomal protein S4 in Proteobacteria**. The phylogenetic tree for *Proteobacteria *(except *Alphaproteobacteria*) was constructed from 1000 maximum-likelihood inferences and rooted using *Deinococcus-Thermus *and *Thermotogae *as outgroups. Branches that are monophyletic with respect to a class or order are collapsed with the number of taxa in the branch given in parentheses. Node label are bootstrap proportions estimated from 5000 replicates. The scale bar represents one change per site.

To test this hypothesis, we performed a phylogenetic analysis of the sequences in the C-(I), C-(II), and C-(III) groups relative to the C+ sequences. The first group, C-(I), includes the S4 sequences from most of *Betaproteobacteria *and all of *Gammaproteobacteria*. The remaining *Betaproteobacteria *are all of the C+ type, and, interestingly, the C-(I) *Betaproteobacteria *show a gradual loss of the four cysteine residues from two to one and, finally, to zero. The *Gammaproteobacteria *also show a distribution of two, one, or zero cysteine residues. The C-(I) sequences have also lost a three residue turn (the second red block in Figure 1) compared to all of the other variants of S4. Figure 2 shows a maximum-likelihood (ML) reconstruction of the phylogenetic history of S4 in *Proteobacteria *except *Alphaproteobacteria*, which lack the seven residue indel. In the tree, *Beta- *and *Gammaproteobacteria *share a common ancestor containing the C+ variant of S4 and the root of the *Proteobacteria *also appears to have been a C+ type S4. The most parsimonious explanation for the origin of the C-(I) form appears to be that a single evolutionary event, characterized by the deletion of the three residue turn and loss of the zinc-binding motif, occurred in the *Betaproteobacteria *lineage and was inherited monophyletically by the descendant *Betaproteobacteria *and the *Gammaproteobacteria*.

The C-(II) group contains all and exclusively sequences from the *Epsilonproteobacteria*. These sequences do contain the three residue turn that the C-(I) *Beta- *and *Gammaproteobacteria *sequences are missing. Additionally, the pattern of disruption in the zinc-binding motif is markedly different from the C-(I) group. In C-(II) sequences, the four cysteine residues are consistently replaced by two glutamic acid residues, one arginine residue, and one serine residue, as opposed to the variety of residues seen in C-(I). differences in both the pattern of indels and of motif disruption suggest an independent origin for the C-(II) form and phylogenetic analysis supports this interpretation. In the tree shown in Figure 2, *Epsilonproteobacteria *branches outside of the C-(I) group, appearing to diverge near the root of *Proteobacteria*. The low bootstrap values at higher branch points do cast uncertainty as to whether *Epsilonproteobacteria *diverged from a common *Proteobacteria *ancestor or directly from the bacterial root. In either case, however, the C-(II) sequences would be a result of an independent mutation event in an ancestral C+ form that occurred after the divergence of the *Epsilonproteobacteria *lineage. Conservation of the "EXXE...RXXXXS" motif suggests that a salt bridge may have replaced the zinc finger as a structural element in the C-(II) S4 sequences, and threading of an epsilonproteobacterial sequence onto the *T. thermophilus *crystal structure of S4 confirms that the residues would be properly oriented.

The final C- type containing the seven residue indel, C-(III), is made up of S4 sequences from a subset of *Spirochetes*: the genus *Leptospira*. All other *Spirochetes *currently sequenced lack the indel in r-protein S4. C-(III) sequences have a zinc disruption pattern of "VXXM...LXXXXS" or "VXXM...FXXXXF" and do have the three residue turn missing in C-(I). Additionally, there are numerous sequences signatures separating the C-(III) sequences from those in either group C-(I) or C-(II). Phylogenetically, these sequences appear to branch directly from the root of the C+ form, no further relationships can be resolved. Since the C-(III) group appears to monophyletically descend from an ancestral C+ form, we consider that it too was an independent evolution of zinc disruption in S4.

All of the remaining S4 C- sequences lack the seven residue indel and both pairs of cysteine residues. C-(IV), the largest C- group, consists of r-protein S4 sequences from a wide variety of bacteria: *Actinobacteria*, *Alphaproteobacteria*, *Aquificae*, *Bacilli*, *Bacteroidetes*, *Betaproteobacteria*, *Chlamydiae*, *Chlorobia*, *Chloroflexi*, *Cyanobacteria*, *Deinococcus-Thermus*, *Deltaproteobacteria*, *Fusobacteria*, *Mollicutes*, *Planctomycetes*, *Spirochaetes*, and *Verrucomicrobia*. The N-terminal domains of the C-(IV) sequence are much less conserved than the C+ form, having an average percent identity of 36%, and do not contain any characteristic sequence or structural signatures by which they could be further classified.

A small number of *Clostridia *sequences (17) constitutes the last defined type, C-(V). These C- sequences lack the seven residue indel, but are different from C-(IV) sequences (and all other S4 sequences) in that they are missing a "PGXHG" motif starting at residue 38. This motif is highly conserved in the other S4 sequences and is unambiguously alignable across all other groups. In C-(V) *Clostridia *sequences, this region is 2–4 residues shorter and can not be reliably aligned to the other types. All but one of the S4 sequences in this group are from genomes that also contain a C+ type S4.

### Phylogenetic reconstruction of S4's evolutionary history

**Figure 3:**
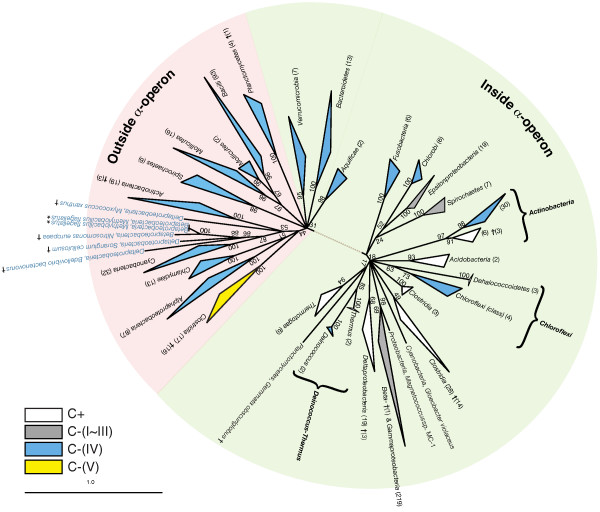
**Consensus unrooted phylogenetic tree of ribosomal protein S4**. The tree was constructed from 1000 maximum-likelihood inferences. Node labels are bootstrap proportions estimated from 5000 replicates. Branches that are monophyletic with respect to a phylum or class and also with respect to a sequence classification have been collapsed, parentheses give the number of sequences in the branch. Colors indicate the sequence classifications: (white) C+, (gray) C-(I) – C-(III), (blue) C-(IV), and (yellow) C-(V). Sequences from genomes with multiple *divergent *copies of an S4 gene are marked with a (†) dagger. The two sequences marked with an (*) asterisk are identical copies resulting from large-scale genome duplication.

The evolutionary history of S4 was analyzed using a ML phylogenetic reconstruction of all the sequences of r-protein S4 and its paralogs obtained from the 660 genomes, as described in Methods. Figure 3 shows an unrooted phylogenetic tree obtained from a consensus of 1000 ML trees. Like many phylogenetic reconstructions using a large number of sequences, branch points above the bacteria phyla level are difficult or impossible to reliably determine [28] and most branches appear to radiate from a few ancestral points in our consensus tree. Trees of the C-terminal RNA binding domain and the N-terminal bacterial specific domain were also generated separately using the same method (data not shown). The C-terminal tree had similar branchings as the tree shown in Figure 3, but with fewer well-supported branches near the bacterial root. The N-terminal domain, however, is too short to draw any reliable conclusions regarding its relative contribution to the phylogenetic signal.

The consensus phylogenetic tree of the entire protein shows good agreement with the classifications of S4 that we introduced earlier. It is roughly divided into two central foci, one representing the C+ form (white) and the other the C-(IV) form (blue), although a few C-(IV) lineages branch within the C+ half. C-(I), C-(II), and C-(III) (gray) are recent, independent mutations of an original C+ form (discussed above), and we treat them as C+ for the remainder of the discussion. The C-(V) form (yellow) is a monophyletic branch descending from the C-(IV) root.

Within the C+ branch of the tree, three bacteria phyla are monophyletic with high support values and yet contain both C+ and C-(IV) forms: *Actinobacteria*, *Chloroflexi *and *Deinococcus-Thermus*. In each case, there are two branches descending from the phylum that are monophyletic, one with respect to C+ and one to C-(IV). Despite the sequences in each C+ branch containing the seven residue indel characteristic of the C+ group and sequences in each C-(IV) branch lacking the indel, the branches have a higher average sequence identity (56%, 46%, 60%, respectively) than in general would be expected for a C+ and a C-(IV) group (~40%). We therefore consider it likely that these are real phylogenetic branches and not artifacts, particularly for *Actinobacteria *and *Deinococcus-Thermus*.

Also within the C+ branch, the C-(IV) *Chlorobi *and C+ *Epsilonproteobacteria *group together, with an average percent identity of 50%. However, the *Chlorobi *sequences have nearly as high average percent identity with C-(IV) groups (46%) and the support value of 52 is fairly low, so this grouping may be a reconstruction artifact.

Comparing the S4 phylogenetic tree with the classical bacterial phylogeny, many bacterial groups show good agreement with the tree at the phyla level: The phyla *Aquificae*, *Bacteroidetes*, *Chlamydiae*, *Tenericutes *(*Mollicutes*) and *Verrucomicrobia *in the C-(IV) branch and *Acidobacteria*, *Chlorobi*, *Chloroflexi*, *Deinococcus-Thermus*, *Fusobacteria*, and *Thermotogae *in the C+ branch are all monophyletic with high support values in the tree. A few other groups, most notably the *Proteobacteria *and the *Firmicutes*, are monophyletically supported at the class level. *Proteobacteria *classes *Beta/Gamma-*, *Delta-*, and *Epsilonproteobacteria *independently meet at the root of the C+ branch, while *Alphaproteobacteria *originates in the C-(IV) branch. In the *Firmicutes*, *Clostridia *(non-paralogs) branches from C+ while *Bacilli *branches from C-(IV). The remaining bacteria phyla, *Actinobacteria*, *Cyanobacteria*, *Planctomycetes*, and *Spirochaetes*, have more convoluted branching patterns, with members branching in either the C+ or C-(IV) group with little regard for classical phylogeny. Disagreement with the classical bacterial phylogeny is an indication that a process more complex than standard vertical inheritance occurred with r-protein S4 during bacterial evolution.

### Identification of paralogous and duplicated S4 genes

A key element that led to the identification of HGT and gene duplication with DGL in previous bacterial r-proteins was the analysis of genomes containing multiple copies of the r-protein genes [14]. Among the 660 bacterial genomes in our study, 26 organisms from the groups *Clostridia*, *Betaproteobacteria*, *Deltaproteobacteria*, *Gammaproteobacteria*, *Actinobacteria*, *Spirochaetes *and *Planctomycetes *possess more than one copy of the S4 gene (marked with a dagger symbol on the tree in Figure 3). Most have two copies, as shown in Table 1, and normally one copy is a C+ form and the other a C-(IV) or C-(V). Two *Clostridia*, *Alkaliphilus metalliredigens *and *Clostridium acetobutylicum*, have three copies of the S4 gene, both have one C+ and two C-(V) variants. The genomes of *Methylobacillus flagellatus*, *Psychromonas ingrahamii*, and *Leptospira borgpetersenii *do not match the above pattern in that both genes are of the same type. However, in each of these three cases the sequence identities of the two copies are extremely high, 100%, 99%, and 100%, respectively. These cases are undoubtedly recent gene duplication events. In fact, *P. ingrahamii *and *L. borgpetersenii *have duplicated a large segment of their conserved operon cluster. *M. flagellatus *is known to have a large 140 kbp repeat in its genome [29], this repeated region contains the S4 gene.

**Table 1:** Genomes containing multiple copies of the S4 gene

Organism Name	Taxon	# copies	PID(%)	Classification	Other Zn-ribbon duplicates^*a*^
*Frankia *sp. EAN1pec	Actinobacteria	2	40.57	**C+^*b*^**; C-(IV)	
*Salinispora arenicola *CNS-205		2	36.79	**C+**; C-(IV)	S14, L33, L28^*c*^, L31, L32
*Salinispora tropica *CNB-440		2	36.32	**C+**; C-(IV)	S14, L33, L28^*c*^, L31, L32
*Methylobacillus flagellatus *KT	*β*-proteobacteria	2	100.0	both C-(IV)	L36
*Nitrosomonas europaea *ATCC 19718		2	36.62	**C+**; C-(IV)	
*Psychromonas ingrahamii *37	*γ*-proteobacteria	2	99.51	both **C-(I)**	L36^*d*^
*Bdellovibrio bacteriovorus *HD100	*δ*-proteobacteria	2	39.35	**C+**; C-(IV)	
*Myxococcus xanthus *DK 1622		2	36.41	**C+**; C-(IV)	S14, L33, L28^*c*^
*Sorangium cellulosum *'So ce 56'		2	42.45	**C+**; C-(IV)	
*Alkaliphilus metalliredigens *QYMF	Clostridia	3	38.53^*e*^	**C+**; C-(V)^*f*^	
*Alkaliphilus oremlandii *OhILAs		2	40.00	**C+**; C-(V)	
*Clostridium acetobutylicum *ATCC 824		3	38.61^*e*^	**C+**; C-(V)^*f*^	
*Clostridium botulinum *A str. ATCC 3502		2	38.79	**C+**; C-(V)	
*Clostridium botulinum *A3 str. Loch Maree		2	37.38	**C+**; C-(V)	
*Clostridium botulinum *A str. ATCC 19397		2	38.79	**C+**; C-(V)	
*Clostridium botulinum *A str. Hall		2	38.79	**C+**; C-(V)	
*Clostridium botulinum *B1 str. Okra		2	38.79	**C+**; C-(V)	
*Clostridium botulinum *F str. Langeland		2	39.25	**C+**; C-(V)	
*Clostridium kluyveri *DSM 555		2	39.25	**C+**; C-(V)	
*Clostridium novyi *NT		2	40.38	**C+**; C-(V)	
*Clostridium perfringens *str. 13		2	38.79	**C+**; C-(V)	
*Clostridium perfringens *ATCC 13124		2	38.79	**C+**; C-(V)	
*Clostridium perfringens *SM101		2	38.79	**C+**; C-(V)	
*Leptospira borgpetersenii *serovar	Spirochaetes	2	100.0	both **C-(III)**	S14, L36^*g*^
Hardjo-bovis L550					
*Gemmata obscuriglobus *UQM 2246	Planctomycetes	2	40.95	**C+**; C-(IV)	

^*a*^Observations based on annotation.

^*b*^Classification written in bold indicates that it is inside the *α*-operon.

^*c*^The duplicates of the zinc-binding r-proteins S14, L28, L33 sit together in the genome.

^*d*^Resulting from the duplication of the whole *α*-operon.

^*e*^Average value of the percent identities of the two pairs of in-operon and out-of-operon copies.

^*f *^*A. metalliredigens *and *C. acetobutylicum *have 2 copies of the C-(V) form.

^*g*^Resulting from the duplication of the entire s10-spc and *α*-operon.

Usually in a case of two divergent copies of a gene in a genome, one copy is the original and the other a paralog, either from an ancient gene duplication or from an HGT event. Without experimental evidence of activity, determining which is the active gene and which the paralog can often be problematic. In the case of r-protein S4, however, the genome content can provide evidence to make a determination: many ribosomal protein genes in bacteria are known to be located in conserved gene clusters. The gene for S4 is usually located in a cluster along with the genes for ribosomal proteins S13, S11, and L17 and the gene for the RNA polymerase alpha subunit, which together are known as the *α*-operon because they are co-regulated in *E. coli*. If two copies of the S4 gene are present in a genome with one copy inside the *α*-operon and the other outside it, we assume the copy inside the *α*-operon is the original form and the other the paralog. In every genome containing *two divergent *S4 genes, the C+ form is located in the *α*-operon and the C-(IV) or C-(V) form outside. Using the above criteria, we conclude that the C+ form is the original S4 sequence and the C- form the paralog in these genomes. It then remains to determine the origin of the paralogous C- sequences, whether by HGT or gene duplication.

One clear-cut case of HGT appears to have occurred in the *Proteobacteria*. One *Beta- *and three *Deltaproteobacteria *have S4 paralogs that group within the C-(IV) branch of the phylogenetic tree in Figure 3 and C+ genes in the *α*-operon. Since the vast majority of *Beta- *and *Deltaproteobacteria *have only a single C+ copy of S4, we consider it unlikely that this pattern resulted from an ancient gene duplication that was lost in all *Beta- *and *Deltaproteobacteria *except these four organisms. Given the high support values near the branch with *Cyanobacteria*, we find it more likely that these organisms obtained the gene through a horizontal transfer from *Cyanobacteria*, although the S4 sequence is not similar enough to any available sequences for the specific source organism to be determined. *Betaproteobacteria *species *M. flagellatus*, which possesses only two copies of C-(IV) outside of the *α*-operon, also groups nearby in the tree and also likely received its C-(IV) S4 gene from *Cyanobacteria *before its large-scale genome duplication occurred. It must have lost its original C+ gene subsequent to the HGT, as it is no longer present in the *α*-operon.

**Figure 4:**
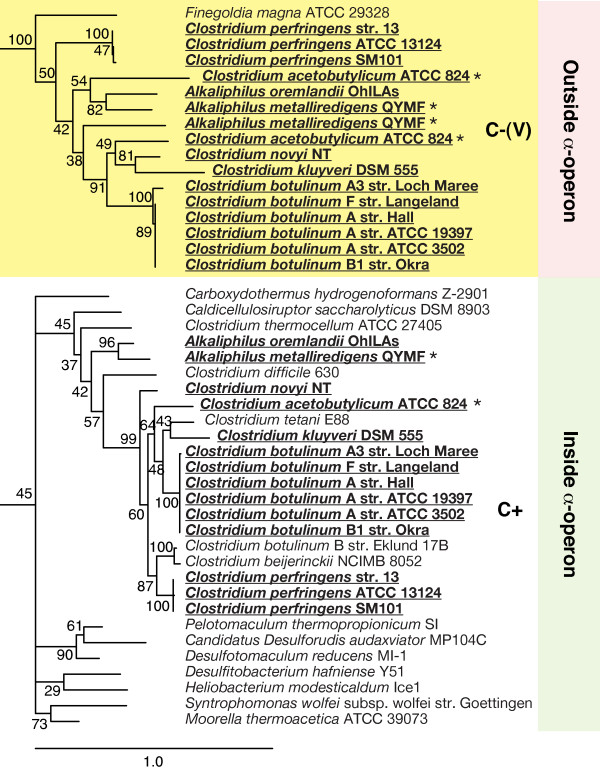
**Expansion of Clostridia branches of the consensus S4 phylogenetic tree**. The C+ and C-(V) *Clostridia *branches are highlighted white and yellow, respectively. Sequences from organisms with multiple S4 genes are in bold underline. Sequences from the two genomes with three S4 genes are additionally marked with an (*) asterisk.

Another example of probable HGT, albeit with a more complex pattern, is seen in the *Clostridia*. All *Clostridia *except one, *Finegoldia magna*, contain the gene for the C+ form of S4 in the *α*-operon. Fifteen *Clostridia *also contain a gene for the C-(V) form of the S4 sequence. Figure 4 shows an expansion of these two *Clostridia *branches from the consensus phylogenetic tree. In the C+ branch, all of the organisms with multiple copies of S4 are descended from a single branch. If we assume that the C+ form represents the vertical phylogeny in this group, then the pattern is consistent with either a single HGT event in the ancestor of the *Clostridium *and *Alkaliphilus *genera with later differential loss in a few branches; or with three later HGT events, one for *Alkaliphilus*, a second for *Clostridium perfringens*, and a third for the branch containing the organisms *Clostridium botulinum/kluyveri/acetobutylicum/novyi*. However, two organisms possess three copies of the S4 gene, and the percent identities between the two C-(V) copies are 60.3% and 54.3% for *A. metalliredigens *and *C. acetobutylicum*, respectively. So there may have been an additional recent HGT of the transferred C-(V) gene. The support values are too low to allow a determination the source of this recent HGT, but additional genomes of related organisms could shed light on the history of the C-(V) form of S4.

**Figure 5:**
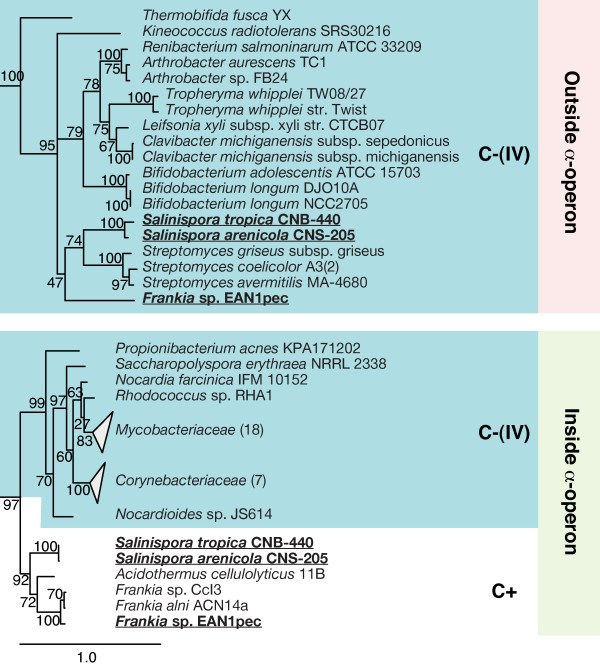
**Expansion of Actinobacteria branches of the consensus S4 phylogenetic tree**. The C+ and C-(IV) *Actinobacteria *branches of S4 are highlighted white and blue, respectively. Sequences from organisms with multiple S4 genes are in bold underline.

In the two remaining lineages with genomes containing multiple S4 genes, multiple occurrences are relatively rare. Of the four *Planctomycetes *genomes available, one contains both the C+ gene in the *α*-operon and C-(IV) out of it while the other three contain only C-(IV) out of the operon. The low number of available *Planctomycetes *genomes sequenced makes it impossible to reconcile the origin of the paralog using a parsimony argument. In the *Actinobacteria*, the three genomes with two copies of the S4 gene can be accounted for by two recent HGT events (see Figure 5), one in the *Salinispora *genus and the other in the species *Frankia *sp. EAN1pec. Both of these paralogous genes appear to have originated in a *Streptomyces *source. The two *Salinispora *species also have acquired paralogs of five other zinc-binding r-proteins.

### Regulation of C- S4 paralogs

To fully understand the evolutionary pressure giving rise to paralogous genes, it is helpful to know their regulation mechanism, especially when the two copies have similar functions. For the previously known zinc-binding ribosomal proteins, experiments on specific bacteria have shown that the paralogs can be turned on and off in response to zinc conditions. In *B. subtilis *(S14, L31) [17], *M. tuberculosis *(S14, S18, L28, L33) [19], and *S. coelicolor *(S14, L28, L31, L32, L33A, L33B, L36) [18], the C- paralogs were found to be expressed only under low zinc conditions. Their regulation was controlled by the zinc uptake regulator (Zur) transcription factor, except for L33B and L36 in *S. coelicolor*, the regulation of which was controlled by a sigma factor (*σ*^*R*^).

The established Zur binding-sites from *Actinobacteria*, *Bacilli *and *Proteobacteria *are AT-rich palindromes found upstream from the genes being regulated [15,18,19]. Using a profile of Zur binding motifs from these bacterial groups, we searched the 26 genomes containing multiple copies of the S4 gene (from *Actinobacteria*, *Clostridia *and *Beta/Deltaproteobacteria*) for candidate Zur binding sites (see Methods). We were able to identify Zur binding sites upstream of the gene cluster of r-proteins L33, S14 and L28 and upstream of the paralogous genes of both L31 and L32 in *Salinispora arenicola *and *Salinispora tropica*, but no binding sites were found near the paralogous S4 genes. Neither were Zur binding sites found near ribosomal protein paralogs in the remaining genomes. Unfortunately, a Zur binding motif has not yet been reported for *Clostridia*, which comprises most of the genomes with paralogous copies of the S4 gene. Therefore, we can not exclude the possibility that the paralogous copies of S4 in *Clostridia *are regulated by Zur binding to a motif different from any in our profile.

However, according to gene expression data from two separate genomic-scale gene expression experiments in *C. acetobutylicum *[30] and *Clostridium novyi *[31], the paralogous C-(V) genes are not expressed under normal growth conditions but are up-regulated during sporulation. This leaves open the possibility that the C-(V) genes are related to some aspect of ribosomal function during sporulation and not used to regulate the zinc environment in *Clostridia*. If the C-(V) S4 proteins are indeed incorporated into ribosomes in clostridial spores, it would be interesting to examine any changes to these ribosomes, such as altered structure or changes in the assembly process.

### Comparison of genome content near S4

**Figure 6:**
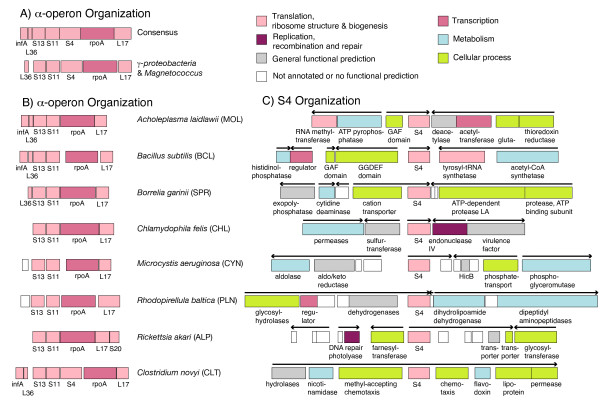
**Genomic content near S4 and the α*-operon***. Shown are a representative sample of the genomes. A) Consensus genome context of the S4 gene in the *α*-operon across most bacterial groups. B & C) Context of the *α*-operon and S4, respectively, in bacterial groups where an S4 gene is located outside the *α*-operon. Phyla abbreviations are given in parentheses. The lengths of the genes are to scale and gene are color-coded according to COG (clusters of orthologous groups) functional categories.

Having used genome context in the analysis of several cases of horizontal transfer, we next examined the genome regions near S4 and the *α*-operon in the genomes of the bacteria without multiple copies looking for conserved patterns. Overall, the organization of the *α*-operon and nearby genes is highly conserved across a large number of bacterial groups. Many of the genomes have the conserved consensus gene cluster shown in Figure 6A, containing genes for initiation factor A (infA), L36, S13, S11, S4, RNA polymerase subunit A (rpoA) and L17. Variations are mainly seen in *Gammaproteobacteria *and *Magnetococcus*, which do not have infA near the cluster. Intriguingly, genes for *both *the C+ and C-(IV) forms of S4 can be found in the *α*-operon (green background in Figure 3). In fact, eight phyla have the gene for the C-(IV) form located in the *α*-operon, including all three of the phyla containing closely branching C+ and C-(IV) forms (*Actinobacteria*, *Chloroflexi *and *Deinococcus-Thermus*). Five other phyla, *Aquificae*, *Bacteroidetes*, *Chlorobi*, *Fusobacteria*, and *Verrucomicrobia*, contain only the C-(IV) form in the operon.

The remaining bacterial genomes, still covering a diverse set of bacteria, contain only an S4 gene of the C-(IV) form that is not located within the *α*-operon (red background in Figure 3). Figure 6B and 6C shows examples of the genomic context of the *α*-operon and the S4 gene, respectively, in these genomes. The organization of the genes remaining in the *α*-operon is unperturbed, but the context around the S4 gene is variable. Conservation of organization near the S4 gene can only be seen at the level of order or family; no correlations with the organization of any other genes could be detected at higher levels of taxonomy. When not located in the *α*-operon, the gene for S4 appears to be quite mobile.

## Discussion

### The ancestral form of S4 in the bacteria

**Figure 7:**
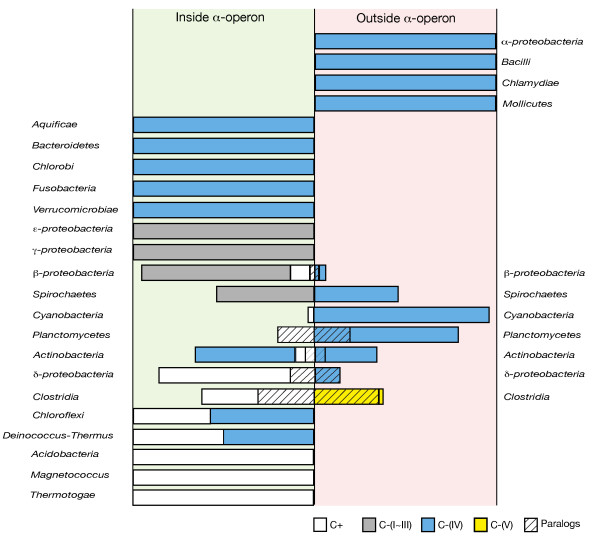
**Occurrence of different types of S4 throughout the bacterial phylogeny**. Length of each bar is scaled to match the percentage of the corresponding type of sequences in the group. Colors indicate the sequence classifications: (white) C+, (gray) C-(I) – C-(III), (blue) C-(IV), and (yellow) C-(V). Boxes with hatch marks are paralogous sequences, which are of different types inside (green background) or outside (red background) the *α*-operon.

Given the widespread occurrence of C-(IV) genes within the *α*-operon (see Figure 7), one must question the hypothesis that the C+ zinc-binding form of S4 is ancestral in the bacteria. If the C-(IV) form were a result of a single ancient gene duplication of a C+ gene, one would have expected to find nearly all of C-(IV) genes located outside of the *α*-operon. Instead, five classical bacteria phyla contain exclusively the C-(IV) gene in the *α*-operon. Moreover, three bacteria phyla contain monophyletic branches of both the C+ and C-(IV) genes, each organized in the typical *α*-operon style. Although it is known that horizontally transferred genes can replace their native copies in the genome, so called *in situ *gene displacement [32], such occurrences are still thought of as exceptions rather than the rule. The number of *in situ *displacements required to achieve the current distribution of C-(IV) genes in the *α*-operon would require replacement events of a much higher frequency or different character than that previously reported.

We propose instead that neither C+ nor C-(IV) is the sole ancestral form of S4 and interpret the data as implying the presence of both forms during the time when the bacterial lineages were diverging. The developing bacterial lineages would have sampled S4 genes from the bacterial pool according to some unknown criteria, perhaps related to the local environment (*e.g*., thermophilic organisms acquiring the zinc-binding form for added stability). While this sampling would have been functionally equivalent to HGT with *in situ *gene displacement, in that the gene order would be maintained, it would not have necessarily been mechanistically related to the process by which HGT occurs today.

Additional support for the existence of innovation sharing within gene pools comes from signatures in the S4 protein that were reported by Roberts *et al*. [2] to distinguish the bacterial and archaeal/eukaryal lineages. S4 proteins from both archaea and bacteria possess the RNA binding C-terminal domain, but have an N-terminal architecture distinct to each domain of life. Furthermore, the archaeal version of the *α*-operon is organized with S4 preceding S11 (S13-S4-S11), as opposed to S11 proceeding S4 as in bacteria (S13-S11-S4). Clearly, large-scale evolutionary changes occurred in S4 after (or at) the *Bacteria *and *Archaea *divergence, and yet the signatures are unvarying within each domain. Excluding the possibility that all extant bacteria can trace their vertical ancestry to a single individual cell and all extant archaea to another single cell, the respective organism pools at the time must have been able to efficiently share genes in an *in situ *manner that allowed the homogenization of the bacterial pool. This is the same evolutionary process required to support both a C+ and a C-(IV) form of the S4 gene in the bacterial pool.

### Origin of S4 outside the α-operon

If, as suggested above, a bacterial pool allowed both the C+ and C-(IV) forms of the S4 gene to be brought into the genome *in situ *as needed, the question arises as to the origin of the C-(IV) gene outside of the *α*-operon in genomes where it is the sole copy. We propose that this organization is the result of HGT of the C-(IV) gene into C+ genomes after the phyla had diverged from the bacterial gene pool and the *in situ *evolutionary dynamic had slowed. Loss of the original C+ gene would have then allowed a reduction in zinc use without perturbation to growth of the organisms.

**Figure 8:**
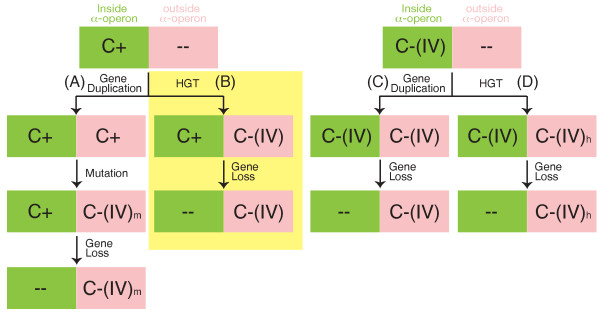
**Possible evolutionary pathways**. Four possible evolutionary pathways (A-D) that result in the observed pattern of a single C-(IV) gene outside the *α*-operon.

Figure 8 depicts the four possible evolutionary paths (labeled A-D) starting from either a C+ or C-(IV) gene inside the *α*-operon and ending with a single C-(IV) type gene outside the *α*-operon. Path A involves a gene duplication of a C+ type, mutation of the C+ type into a C-(IV) type, and finally loss of the original C+ gene. This path is ruled out for two reasons: first, no duplications of C+ S4 genes were observed in any of the 660 genomes studied, and second, the path depends on an unlikely set of mutation events. The C-(IV) genes outside the *α*-operon are indistinguishable in sequence from the C-(IV) genes inside, including the loss of a characteristic seven residue indel. The probability of an independent mutational deletion of seven residues from a C+ gene leading to the exact same indel pattern as in the pre-existing C-(IV) gene is low. Additionally, there are other sequences signatures, such as residue 15 (in the RRXG motif) being glutamic acid in C+ and leucine/phenylalanine in C-(IV) and residue 21 being leucine in C+ and glycine/proline in C-(IV), that support a common origin for all of the C-(IV) sequences.

Path B specifies HGT of a C-(IV) gene into a genome containing the C+ type in the *α*-operon followed by loss of the C+ gene. Evidence supporting path B comes from the pattern of S4 HGT events presented in Results. The evolutionary history of S4 contains several relatively recent horizontal transfers, as supported by our analysis of genomes containing multiple copies of the S4 gene. In each of these cases, a C-(IV) gene was transferred into a genome with the C+ form of the S4 gene in the *α*-operon. Additionally, in two instances there was loss of the original C+ gene following the HGT of a C-(IV) gene, the clostridium *F. magna *and the betaproteobacterium *M. flagellatus*, exactly as prescribed in path B.

Both of the remaining paths, C and D, start with a C-(IV) gene and involve later acquisition of an additional C-(IV) gene either through duplication of the original or HGT, respectively. Our analysis found neither duplications nor horizontal transfers in any genome with the C-(IV) gene in the *α*-operon, although the sample size of known HGT events is low. Furthermore, there is phylogenetic evidence that some of the groups now containing only a C-(IV) gene outside of the *α*-operon are descended from lineages originally containing the C+ gene, which would preclude paths C and D. For example, all *Alphaproteobacteria *contain only the C-(IV) gene outside of the *α*-operon, while all other *Proteobacteria *have the C+ form (or recent variations thereof) in the *α*-operon. Even the genome of the unclassified proteobacterium *Magnetococcus sp. MC-1*, which is phylogenetically closest to the *Alphaproteobacteria *[33,34], contains the gene for the C+ form of S4 in the *α*-operon. Thus, the *Proteobacteria *phylum likely contained the C+ gene originally.

The above arguments provide support for our hypothesis that C+ was the original form of the S4 gene in the branch of the tree containing C-(IV) outside the *α*-operon (red background in Figure 3) and that these branches received the C-(IV) gene through HGT. The original source of the C-(IV) S4 gene must have been one of the phyla containing C-(IV) natively, *i.e*., one with the C-(IV) gene in the *α*-operon, but once the S4 gene made the transition from an operon gene to a standalone gene it may have become more readily transferable. Later HGT events may therefore have originated from organisms having already received prior transfers. From the phylogenetic tree in Figure 3 the best candidate phyla for the original source are *Aquificae*, *Bacteroidetes *and *Verrucomicrobia*, but the low support values near the radiating points in the tree leave a great deal of ambiguity as to the exact source. Given the strict presence of the C-(IV) form and absence of an S4 gene in the *α*-operon in the groups, it appears that both the HGT events and native gene losses are likely ancient.

### Evolutionary pressure and the loss of zinc binding in ribosomal proteins

Insight into an evolutionary process comes from not only describing the mechanism of change, but also the pressures behind the change. As discussed earlier, seven other r-proteins have been reported to bind zinc and to have evolutionary histories disrupted in a similar pattern to what we have reported for S4. Table 2 shows the occurrence of C+, C-, or both C+ and C- genes of these r-proteins in the major bacterial groups (a complete breakdown by organism is available as Additional File 1). C+ was reported as being the ancestral form of these r-proteins [14] and, if that is indeed the case, it is clear from the distribution that large groups have developed either the ability to do without zinc for specific r-proteins or to switch to C-paralogs under low zinc conditions. Specifically, we see characteristic divisions *below *the phyla level, *e.g. Alphaproteobacteria *have replaced the C+ genes for almost all zinc-binding r-proteins with C- genes, *Epsilonproteobacteria *has exclusively C- forms of three, and *Magnetococcus sp. MC-1 *encodes the C- gene for only one.

**Table 2:** Taxonomic distributions of the C +/- ribosomal proteins

	L32	L36	L31	S14	L33	S18	L28	S4
Verrucomicrobiae	-	-	-	-	-	-	-	-
*α*-proteobacteria	+,-	-	-	-	-	-	-	-
Bacteroidetes	+,-	-	-	-	-	-	-	-
Chlamydiae	+	-	-	-	-	-	-	-
Fusobacteria	+	nd	-	-	-	-	-	-
*β*-proteobacteria	-	+,+/-	+,-,+/-	-	-	-	-	+,-
*γ*-proteobacteria	-	+,-,+/-	+,-,+/-	-	-	-	-	-
Chlorobi	+	+	+	-	-	-	-	-
Planctomycetes	nd	-	+	+	-	-	-	-,+/-
Deinococcus	+	+	-	+, -	-	-	-,+/-	-
Cyanobacteria	-	+,-	+,-	+,-	+,-	-	-	+,-
Bacilli	+,-	+	-,+/-	+,-,+/-	-,+/-	-	-	-
Tenericutes (Mollicutes)	+,-	+,-	+	+,-	+	+,-	-	-
*ε*-proteobacteria	-	+	+,-	+	+,-	+,-	-	-
Actinobacteria	+,-	+	+,-,+/-	+,-,+/-	+,-,+/-	+,-,+/-	+,-,+/-	+,-
Chloroflexi	+	+	+,-	+	+,-	+,-,+/-	+,-,+/-	+,-
Spirochaetes	+	+	+,-	+	-	+,-	+,-	+,-
Thermus	+	+	+	+	+	-	-	+
Magnetococcus	+	+	+	+	+	+	-	+
Aquificae	+	+	+	+	+	+	+	-
*δ*-proteobacteria	+	+	+	+	+,-	+	+	+
Clostridia	+	+	+	+	+	+	+	+,+/-
Acidobacteria	+	+	+	+	+	+	+	+
Thermotogae	+	+	+	+	+	+	+	+

The complete data set is given as Additional File 1.

+,- indicates each form comprises at least 10% of the group, but occurs only once in each genome.

+/- indicates at least 10% of the genomes in the group contain both forms. This cut-off removes all minor cases of HGT. nd indicates that the r-protein was not detected.

These observations point to the conclusion that some bacteria evolved to use C- variants of the zinc-binding ribosomal proteins (including S4) to regulate the zinc economy of the cell. Whether this lower zinc usage was a response to a change in the zinc conditions in the environment or whether some other change in the environment (such as lower temperature) caused the decreased need for zinc is still unclear.

## Conclusion

The cellular information processing system is generally believed to be much less subject to the influences of HGT than other genetic systems. While recent metagenomic studies have not reported any reliable HGT events for the ribosome among the three domains of life, examples of disagreement with the UPT among the bacterial versions of seven zinc-binding r-proteins S14, S18, L28, L31, L32, L33 and L36 have been well documented [12,14]. According to our study of 660 bacterial genomes, the bacterial version of the universal r-protein S4, shares similarities with these seven proteins, namely they all have two different versions of the sequence, zinc-binding (C+) and non-zinc-binding (C-), and their evolutionary histories all show patterns of disagreement with the standard UPT.

The evolutionary history of r-protein S4 reconstructed here shows that S4 was subject to horizontal transfer throughout the history of the bacterial lineages. Recent HGT of the standard character was observed along with other less well-defined evolutionary dynamics of ancient origin. We propose wide-spread sampling of ancestral C+ and C- forms of the S4 gene from a bacterial gene pool as a possible explanation, but definitive proof of such an ancient event cannot be easily obtained. The present study was only possible given the large number of available bacterial genomes, and perhaps additional genomes of other diverse bacterial lineages would provide additional evidence for or against this proposition. Experiments detailing the purpose and regulation of paralogous S4 genes in *Clostridia *also may shed light on the differences between the C+ and C- forms.

In more practical terms, it should now be understood that even "core" proteins can have a more complex evolutionary history than can be explained by vertical inheritance. One recent study attempting to reconstruct an organismal tree of life included S4 in a concatenated gene tree [35]. Although the authors did attempt to remove genes subject to HGT, none was detected in the case of S4. It is clear from the present study that doing so is not always a simple proposition. Accurate evolutionary relationships for S4 were only uncovered with extensive coverage of the bacterial tree along with heavy use of genome content. Others have shown that concatenated genes trees may lack resolution [36], and this may be a direct result of mixing genes with different complex relationships, like the one reported here for S4.

## Methods

### S4 sequence acquisition and alignment

The analysis was based all of the complete bacterial genomes available at the time in NCBI GenBank. Additionally, in order to provide further data for a few poorly represented phyla, draft genomes from three *Planctomycetes*, five *Fusobacteria*, and six *Verrucomicrobia *were obtained from the Joint Genome Institute, as identified through the Genome OnLine Database (GOLD) [37]. A complete list of the genomes used is provided as Additional File 1.

To find S4 sequences and paralogs in the genomes, a non-redundant sequence profile was constructed as described in Sethi *et al*. [38] starting with annotated S4 sequences from the Swiss-Prot database [39]. This profile was used to do a BLAST search [40] on each genome with a cut-off of 10^-7^. Fragments containing only the C-terminal RNA binding domain were removed. Sequence were classified as C+ or C- by comparison to annotated sequences and then all sequences of each type were aligned using the ClustalW [41] multiple alignment function. The C+ and C- multiple alignments were combined using the ClustalW profile alignment function and the resulting alignment was hand edited to correct poorly aligned regions. The complete sequence alignment is provided as Additional File 2. All operations were performed within the MultiSeq [42] bioinformatics analysis environment.

### Phylogenetic reconstructions

Maximum likelihood (ML) trees were reconstructed using RAxML version 7.0.4 [43]. A value of 10 was used for the maximum initial rearrangement distance (-i 10) and a value of 25 for the number of rate categories (-c 25). The tree for *Proteobacteria *was calculated using the JTT amino acid model [44] (-m PROTMIXJTT) and the tree for *Bacteria *using the WAG model [45] (-m PROTMIXWAG), as these models gave the best likelihood scores for a given maximum-parsimony tree of the respective alignments. A total of 1000 likelihood searches were performed for each alignment starting from unique, random maximum-parsimony trees (-f d -# 1000). The tree with the highest likelihood score was taken to be the ML tree. A consensus tree was constructed from the ML tree by removing bipartitions found in fewer than 50% of the other most likely trees. Following, 5000 non-parametric bootstrap runs were performed starting with the topology of the ML tree (-b -t *ml.tre *-# 5000) to determine support values for the bipartitions. Support values were mapped onto their corresponding branches in the consensus tree. Sequences from a few genomes (Candidatus *Carsonella ruddii *PV, *Sorangium cellulosum *'So ce 56', *Symbiobacterium thermophilum *IAM 14863, *Petrotoga mobilis *SJ95, *Rubrobacter xylanophilus *DSM 9941, *Myxococcus xanthus *DK 1622, *Clostridium phytofermentans *ISDg) were highly mobile during ML reconstruction (likely long-branch artifacts) and so were excluded from the reconstruction and added afterwards using stepwise maximum-parsimony addition (-f p -t *ml.tre*).

### Zinc regulatory motifs

Zinc regulation protein binding motifs, which are AT rich palindromes on the intergenic region of DNA strand were searched using MEME/MAST [46]. MEME was used to make a position specific substitution matrix (PSSM) based on input palindromes. The input profile of *Actinobacteria *was taken from experimentally determined Zur binding sites in *Mycobacterium tuberculosis *and *Streptomyces coelicolor *[18,19] and the profiles for *Bacillus *group and γ-*proteobacteria *were taken from [15]. Then the resulting matrixes were used as input of MAST to search for other binding sites in the whole genomes. Only those genomes that have paralogs of S4 genes were subjected to this analysis. The three reference profiles mentioned above are provided as Additional File 3, 4, and 5.

## Abbreviations

DGL: differential gene loss; HGT: horizontal gene transfer; indel: insertion/deletion; ML: maximum-likelihood; mRNA: messenger RNA; MSA: multiple sequence alignment; rRNA: ribosomal RNA; r-protein: ribosomal protein; SSU: small subunit; UPT: universal phylogenetic tree.

## Authors' contributions

KC, ER and ZLS conceived the study. KC and ER performed the analysis. KC, ER and ZLS analyzed the data. KC, ER and ZLS wrote the manuscript.

## Supplementary Material

Additional File 1 **Genomes used in the study**. The table contains the list of genomes used along with the occurrence of zinc-binding ribosomal proteins sequences in each. Click here for file

Additional File 2 **S4 sequence alignment**. The sequence alignment contains the full alignment of all S4 sequences used in the study. Click here for file

Additional File 3 **Zur binding site search profiles for Actinobacteria**. The Zur binding site searching profile for *Actinobacteria *contains reference profile we used as seeds and fed into the software MEME/MAST to search for the Zur DNA-binding motifs in other paralogous *Actinobacteria *S4 genes. Click here for file

Additional File 4 **Zur binding site search profiles for Gammaproteobacteria**. The Zur binding site searching profile for *Gammaproteobacteria *contains reference profile used as seeds for the software MEME/MAST to search for the Zur DNA-binding motifs in other paralogous *Proteobacteria *S4 genes. Click here for file

Additional File 5 **Zur binding site search profiles for Bacillus**. The Zur binding site searching profile for *Bacillus *contains reference profile used as seeds for the software MEME/MAST to search for the Zur DNA-binding motifs in paralogous *Clostridia *S4 genes. Click here for file
