# Supplementary material for: Horizontal gene transfer of zinc and non-zinc forms of bacterial ribosomal protein S4
Source: BMC Evol Biol. 2009 Jul 29;9:179. doi: 10.1186/1471-2148-9-179 (PMC3087516; doi:10.1186/1471-2148-9-179)
Supplement: Additional File 1 — Genomes used in the study. The table contains the list of genomes used along with the occurrence of zinc-binding ribosomal proteins sequences in each. [file 1471-2148-9-179-S1.pdf]

Table 1: Sequences used in the study^{a,b,c,d,e}

Taxon	S4	S14	S18	L28	L31	L32	L33	L36
**Bacteria** (663)	**79/609**	**244/484**	**115/558**	**99/566**	**316/426**	**188/373**	**158/539**	**416/175**
**Acidobacteria** (2)	**2/0**	**2/0**	**2/0**	**2/0**	**2/0**	**2/0**	**2/0**	**2/0**
**Acidobacteria (class)** (1)	**1/0**	**1/0**	**1/0**	**1/0**	**1/0**	**1/0**	**1/0**	**1/0**
Acidobacteria bacterium Ellin345	C+	C+	^{h}C+	C+	C+	C+	C+	^{h}C+
**Solibacteres** (1)	**1/0**	**1/0**	**1/0**	**1/0**	**1/0**	**1/0**	**1/0**	**1/0**
Solibacter usitatus Ellin6076	C+	C+	^{h}C+	C+	C+	C+	C+	^{h}C+
**Actinobacteria** (54)	**6/50**	**38/38**	**31/42**	**32/47**	**37/34**	**12/9**	**36/41**	**43/0**
**Actinobacteria (class)** (54)	**6/50**	**38/38**	**31/42**	**32/47**	**37/34**	**12/9**	**36/41**	**43/0**
Acidothermus cellulolyticus 11B	C+	C+	^{h}C+	C+	C+	C+	C+	^{h}C+
Arthrobacter aurescens TC1	^{4}C-	^{4}C-	^{4}C-	^{3}C-	^{4}C-	^{4}C-	^{4}C-	^{h}C+
Arthrobacter sp. FB24	^{4}C-	^{4}C-	^{4}C-	^{3}C-	^{4}C-	^{4}C-	^{4}C-	^{h}C+
Bifidobacterium adolescentis ATCC 15703	^{4}C-	C+	^{4}C-	C+	C+	C+	C+	^{h}C+
**Bifidobacterium longum**								
Bifidobacterium longum DJO10A	^{4}C-	C+	^{4}C-	C+	C+	C+	C+	^{h}C+
Bifidobacterium longum NCC2705	^{4}C-	C+	^{4}C-	C+	C+	C+	C+	^{h}C+
**Clavibacter michiganensis**								
Clavibacter michiganensis subsp. michiganensis NCPPB 382	^{4}C-	^{4}C-	^{4}C-	^{3}C-	^{4}C-	^{4}C-	^{4}C-	^{h}C+
Clavibacter michiganensis subsp. sepedonicus	^{4}C-	^{4}C-	^{4}C-	^{3}C-	^{4}C-	^{4}C-	^{4}C-	^{h}C+
Corynebacterium diphtheriae NCTC 13129	^{4}C-	^{4}C-	^{4}C-	^{3}C-	^{4}C-		^{4}C-	
Corynebacterium efficiens YS-314	^{4}C-	^{4}C-	^{4}C-	^{3}C-	^{4}C-		^{4}C-	
**Corynebacterium glutamicum**								
Corynebacterium glutamicum ATCC 13032		^{4}C-/^{4}C-	^{4}C-/^{4}C-	^{3}C-/^{3}C-	^{4}C-/^{4}C-		^{4}C-/^{4}C-	
Corynebacterium glutamicum ATCC 13032 Bielefeld	^{4}C-							
Corynebacterium glutamicum ATCC 13032 Kitasato	^{4}C-							
Corynebacterium glutamicum R	^{4}C-	^{4}C-	^{4}C-	^{3}C-	^{4}C-		^{4}C-	
Corynebacterium jeikeium K411	^{4}C-	^{4}C-	^{4}C-	^{3}C-	^{4}C-		^{4}C-	
Corynebacterium urealyticum DSM 7109	^{4}C-	^{4}C-	^{4}C-	^{3}C-	^{4}C-		^{4}C-	
Frankia alni ACN14a	C+	C+	^{h}C+/^{4}C-	C+	C+		C+	^{h}C+
Frankia sp. CcI3	C+	C+	^{h}C+	C+	C+		C+	^{h}C+
Frankia sp. EAN1pec	C+/^{4}C-	C+	^{h}C+	C+	C+		C+	
Kineococcus radiotolerans SRS30216	^{4}C-	C+/^{4}C-	^{4}C-	C+/^{3}C-	C+		C+/^{4}C- /^{4}C-	^{h}C+
Leifsonia xyli subsp. xyli str. CTCB07	^{4}C-	^{4}C-	^{4}C-	^{3}C-	^{4}C-	^{4}C-	^{4}C-	^{h}C+
Mycobacterium abscessus	^{4}C-	C+/^{4}C-	^{h}C+/^{4}C-	C+/^{3}C-	C+/^{4}C-		C+/^{4}C-	^{h}C+
**Mycobacterium avium**								
Mycobacterium avium 104	^{4}C-	C+	^{h}C+	C+/^{3}C-	C+		C+/^{4}C-	^{h}C+
Mycobacterium avium subsp. paratuberculosis K-10	^{4}C-	C+/^{4}C-	^{h}C+/^{4}C-		C+/^{4}C-		C+/^{4}C-	^{h}C+
**Mycobacterium bovis**								
Mycobacterium bovis AF2122/97	^{4}C-	C+/^{4}C-	^{h}C+/^{4}C-	^{3}C-/^{3}C-	C+		C+	^{h}C+
Mycobacterium bovis BCG str. Pasteur 1173P2	^{4}C-	C+/^{4}C-	^{h}C+/^{4}C-	^{3}C-/^{3}C-	C+		C+	^{h}C+
Mycobacterium gilvum PYR-GCK	^{4}C-	C+/^{4}C-	^{h}C+/^{4}C-	C+/^{3}C-	C+		C+/^{4}C-	
Mycobacterium leprae TN	^{4}C-	C+	^{h}C+		C+		C+	^{h}C+
Mycobacterium marinum M	^{4}C-	C+/^{4}C-	^{h}C+/^{4}C-	C+/^{3}C-	C+/^{4}C-		C+/^{4}C-	^{h}C+
Mycobacterium smegmatis str. MC2 155	^{4}C-	C+/^{4}C-	^{h}C+/^{4}C-	C+/^{3}C-	C+/^{4}C-		C+/^{4}C-	^{h}C+
Mycobacterium sp. JLS	^{4}C-	C+	^{h}C+	C+/^{3}C-	C+		C+/^{4}C-	^{h}C+
Mycobacterium sp. KMS	^{4}C-	C+	^{h}C+	C+/^{3}C-	C+		C+/^{4}C-	^{h}C+
Mycobacterium sp. MCS	^{4}C-	C+	^{h}C+	C+/^{3}C-	C+		C+/^{4}C-	^{h}C+

Table 1: (cont) Sequences used in the study

Taxon	S4	S14	S18	L28	L31	L32	L33	L36
**Mycobacterium tuberculosis**								
Mycobacterium tuberculosis CDC1551	^{4}C-	C+/^{4}C-	^{h}C+/^{4}C-	C+/^{3}C-/^{3}C-	C+		C+	^{h}C+
Mycobacterium tuberculosis F11	^{4}C-	C+/^{4}C-	^{h}C+/^{4}C-	C+/^{3}C-/^{3}C-	C+		C+	^{h}C+
Mycobacterium tuberculosis H37Ra	^{4}C-	C+/^{4}C-	^{h}C+/^{4}C-	C+/^{3}C-/^{3}C-	C+		C+	^{h}C+
Mycobacterium tuberculosis H37Rv	^{4}C-	C+/^{4}C-	^{h}C+/^{4}C-	^{3}C-/^{3}C-	C+		C+	^{h}C+
Mycobacterium ulcerans Agy99	^{4}C-	C+/^{4}C-	^{h}C+/^{4}C-	C+/^{3}C-	C+/^{4}C-		C+/^{4}C-	^{h}C+
Mycobacterium vanbaalenii PYR-1	^{4}C-	C+/^{4}C-	^{h}C+/^{4}C-	C+/^{3}C-	C+/^{4}C-		C+/^{4}C-	^{h}C+
Nocardia farcinica IFM 10152	^{4}C-	C+/^{4}C-	^{h}C+/^{4}C-	C+/^{3}C-	C+/^{4}C-		C+/^{4}C-	^{h}C+
Nocardioides sp. JS614	^{4}C-	C+	^{h}C+	C+	C+		C+	^{h}C+
Propionibacterium acnes KPA171202	^{4}C-	C+/^{4}C-	^{4}C-	^{3}C-	C+/^{4}C-		C+/^{4}C-	^{h}C+
Renibacterium salmoninarum ATCC 33209	^{4}C-	^{4}C-	^{4}C-	^{3}C-	^{4}C-	^{4}C-	^{4}C-	^{h}C+
Rhodococcus sp. RHA1	^{4}C-	C+/^{4}C-	^{h}C+/^{4}C-	C+/^{3}C-	C+/^{4}C-		C+/^{4}C-	^{h}C+
Rubrobacter xylanophilus DSM 9941	^{4}C-	C+	^{h}C+	C+	^{4}C-	C+	C+	
Saccharopolyspora erythraea NRRL 2338	^{4}C-	C+/^{4}C-	^{h}C+/^{4}C-	C+/^{3}C-	C+/^{4}C-	C+	C+/^{4}C-	^{h}C+
Salinispora arenicola CNS-205	C+/^{4}C-	C+/^{4}C-	^{4}C-	C+/^{3}C-	C+/^{4}C-	C+/^{1}C-	^{1}C-/^{4}C-	^{h}C+
Salinispora tropica CNB-440	C+/^{4}C-	C+/^{4}C-	^{4}C-	C+/^{3}C-	C+/^{4}C-	C+	^{4}C-/^{1}C-	^{h}C+
Streptomyces avermitilis MA-4680	^{4}C-	C+/^{4}C-	^{h}C+/^{4}C-	C+/^{4}C-	C+/^{4}C-/^{4}C-	C+	C+/^{4}C-	^{h}C+
Streptomyces coelicolor A3(2)	^{4}C-	C+/^{4}C-	^{h}C+/^{4}C-	C+/^{3}C-	C+/^{4}C-/^{4}C-	C+	C+/^{4}C-/^{4}C-	^{h}C+
Streptomyces griseus subsp. griseus NBRC 13350	^{4}C-	C+/^{4}C-	^{h}C+/^{4}C-	C+/^{3}C-	C+/^{4}C-/^{4}C-	C+	C+/^{4}C-/^{4}C-	^{h}C+
Thermobifida fusca YX	^{4}C-	C+	^{h}C+	C+	C+	C+	C+	^{h}C+
**Tropheryma whipplei**								
Tropheryma whipplei TW08/27	^{4}C-	^{4}C-	^{4}C-	^{3}C-	^{4}C-	^{4}C-	^{4}C-	^{h}C+
Tropheryma whipplei str. Twist	^{4}C-	^{4}C-	^{4}C-	^{3}C-	^{4}C-	^{4}C-	^{4}C-	^{h}C+
**Aquificae** (2)	**0/2**	**2/0**	**2/0**	**2/0**	**2/0**	**2/0**	**2/0**	**2/0**
**Aquificae (class)** (2)	**0/2**	**2/0**	**2/0**	**2/0**	**2/0**	**2/0**	**2/0**	**2/0**
Aquifex aeolicus VF5	^{4}C-	C+	^{h}C+	^{s}C+	C+	C+	C+	^{h}C+
Sulfurihydrogenibium sp. YO3AOP1	^{4}C-	C+	^{h}C+	C+	C+	C+	C+	^{h}C+
**Bacteroidetes** (13)	**0/13**	**1/12**	**0/13**	**0/13**	**0/13**	**6/4**	**0/10**	**0/7**
**Bacteroidetes (class)** (7)	**0/7**	**0/7**	**0/7**	**0/7**	**0/7**	**4/0**	**0/5**	**0/4**
**Bacteroides fragilis**								
Bacteroides fragilis NCTC 9343	^{4}C-	^{3}C-	^{3}C-	^{2}C-	^{4}C-		^{3}C-	^{3}C-
Bacteroides fragilis YCH46	^{4}C-	^{3}C-	^{3}C-	^{2}C-	^{4}C-	C+		^{3}C-
Bacteroides thetaiotaomicron VPI-5482	^{4}C-	^{3}C-	^{3}C-	^{2}C-	^{4}C-	C+		
Bacteroides vulgatus ATCC 8482	^{4}C-	^{3}C-	^{3}C-	^{2}C-	^{4}C-		^{3}C-	
Parabacteroides distasonis ATCC 8503	^{4}C-	^{3}C-	^{3}C-	^{3}C-	^{4}C-		^{3}C-	
**Porphyromonas gingivalis**								
Porphyromonas gingivalis ATCC 33277	^{4}C-	^{3}C-	^{3}C-	^{3}C-	^{4}C-	C+	^{3}C-	^{3}C-
Porphyromonas gingivalis W83	^{4}C-	^{3}C-	^{3}C-	^{3}C-	^{4}C-	C+	^{3}C-	^{3}C-
**Flavobacteria** (4)	**0/4**	**0/4**	**0/4**	**0/4**	**0/4**	**0/4**	**0/4**	**0/3**
Candidatus Sulcia muelleri GWSS	^{4}C-	^{3}C-	^{3}C-	^{3}C-	^{4}C-	^{4}C-	^{3}C-	^{3}C-
Flavobacterium johnsoniae UW101	^{4}C-	^{3}C-	^{3}C-	^{3}C-	^{4}C-	^{3}C-	^{3}C-	^{3}C-
Flavobacterium psychrophilum JIP02/86	^{4}C-	^{3}C-	^{3}C-	^{3}C-	^{4}C-	^{3}C-	^{3}C-	^{3}C-
Gramella forsetii KT0803	^{4}C-	^{3}C-	^{3}C-	^{2}C-	^{4}C-	^{4}C-	^{3}C-	
**Sphingobacteria** (2)	**0/2**	**1/1**	**0/2**	**0/2**	**0/2**	**2/0**	**0/1**	**0/0**
Cytophaga hutchinsonii ATCC 33406	^{4}C-	^{3}C-	^{3}C-	^{3}C-	^{4}C-	C+		
Salinibacter ruber DSM 13855	^{4}C-	C+	^{4}C-	^{4}C-	^{4}C-	C+	^{3}C-	

Table 1: (cont) Sequences used in the study

Taxon	S4	S14	S18	L28	L31	L32	L33	L36
**Chlamydiae** (13)	**0/13**	**0/13**	**0/13**	**0/13**	**0/13**	**12/0**	**0/11**	**0/8**
**Chlamydiae (class)** (13)	**0/13**	**0/13**	**0/13**	**0/13**	**0/13**	**12/0**	**0/11**	**0/8**
Candidatus Protochlamydia amoebophila UWE25	^{4}C-	^{3}C-	^{3}C-	^{3}C-	^{4}C-			
Chlamydia muridarum Nigg	^{4}C-	^{2}C-	^{3}C-	^{3}C-	^{4}C-	C+	^{4}C-	^{4}C-
**Chlamydia trachomatis**								
Chlamydia trachomatis 434/Bu	^{4}C-	^{2}C-	^{3}C-	^{3}C-	^{4}C-	C+	^{4}C-	^{4}C-
Chlamydia trachomatis A/HAR-13	^{4}C-	^{2}C-	^{3}C-	^{3}C-	^{4}C-	C+	^{4}C-	^{4}C-
Chlamydia trachomatis D/UW-3/CX	^{4}C-	^{2}C-	^{3}C-	^{3}C-	^{4}C-	C+	^{4}C-	^{4}C-
Chlamydia trachomatis L2b/UCH-1/proctitis	^{4}C-	^{2}C-	^{3}C-	^{3}C-	^{4}C-	C+	^{4}C-	^{4}C-
Chlamydophila abortus S26/3	^{4}C-	^{2}C-	^{3}C-	^{3}C-	^{4}C-	C+	^{4}C-	^{4}C-
Chlamydophila caviae GPIC	^{4}C-	^{2}C-	^{3}C-	^{3}C-	^{4}C-	C+	^{4}C-	^{4}C-
Chlamydophila felis Fe/C-56	^{4}C-	^{2}C-	^{3}C-	^{3}C-	^{4}C-	C+		^{4}C-
**Chlamydophila pneumoniae**								
Chlamydophila pneumoniae AR39	^{4}C-	^{2}C-	^{3}C-	^{3}C-	^{4}C-	C+	^{4}C-	
Chlamydophila pneumoniae CWL029	^{4}C-	^{2}C-	^{3}C-	^{3}C-	^{4}C-	C+	^{4}C-	
Chlamydophila pneumoniae J138	^{4}C-	^{2}C-	^{3}C-	^{3}C-	^{4}C-	C+	^{4}C-	
Chlamydophila pneumoniae TW-183	^{4}C-	^{2}C-	^{3}C-	^{3}C-	^{4}C-	C+	^{4}C-	
**Chlorobi** (6)	**0/6**	**0/6**	**0/6**	**0/6**	**6/0**	**5/0**	**0/6**	**5/0**
**Chlorobia** (6)	**0/6**	**0/6**	**0/6**	**0/6**	**6/0**	**5/0**	**0/6**	**5/0**
Chlorobium tepidum TLS	^{4}C-	^{2}C-	^{4}C-	^{3}C-	C+	C+	^{3}C-	^{h}C+
Chlorobium chlorochromatii CaD3	^{4}C-	^{2}C-	^{4}C-	^{3}C-	C+		^{3}C-	^{h}C+
**Chlorobium phaeobacteroides**								
Chlorobium phaeobacteroides BS1	^{4}C-	^{2}C-	^{4}C-	^{3}C-	C+	C+	^{3}C-	^{h}C+
Chlorobium phaeobacteroides DSM 266	^{4}C-	^{2}C-	^{4}C-	^{3}C-	C+	C+	^{3}C-	^{h}C+
Prosthecochloris vibrioformis DSM 265	^{4}C-	^{2}C-	^{4}C-	^{3}C-	C+	C+	^{3}C-	^{h}C+
Pelodictyon luteolum DSM 273	^{4}C-	^{2}C-	^{4}C-	^{3}C-	C+	C+	^{3}C-	
**Chloroflexi** (7)	**2/5**	**7/0**	**5/4**	**5/3**	**5/2**	**7/0**	**3/4**	**6/0**
**Chloroflexi (class)** (4)	**0/4**	**4/0**	**2/4**	**2/3**	**3/1**	**4/0**	**0/4**	**3/0**
Chloroflexus aurantiacus J-10-fl	^{4}C-	C+	^{3}C-	C+/^{3}C-	C+	C+	^{4}C-	^{h}C+
Herpetosiphon aurantiacus ATCC 23779	^{3}C-	C+	^{2}C-		^{4}C-	C+	^{1}C-	
Roseiflexus castenholzii DSM 13941	^{4}C-	C+	^{h}C+/^{3}C-	^{3}C-	C+	C+	^{4}C-	^{h}C+
Roseiflexus sp. RS-1	^{4}C-	C+	^{h}C+/^{3}C-	C+/^{3}C-	C+	C+	^{4}C-	^{h}C+
**Dehalococcoidetes** (3)	**2/1**	**3/0**	**3/0**	**3/0**	**2/1**	**3/0**	**3/0**	**3/0**
Dehalococcoides ethenogenes 195	C+	C+	^{h}C+	C+	^{s}C+	C+	C+	^{h}C+
Dehalococcoides sp. BAV1	^{1}C-	C+	^{h}C+	C+	^{s}C+	C+	C+	^{h}C+
Dehalococcoides sp. CBDB1	C+	C+	^{h}C+	C+	^{1}C-	C+	C+	^{h}C+
**Cyanobacteria** (33)	**1/32**	**1/32**	**0/33**	**0/32**	**1/32**	**0/32**	**14/8**	**24/3**
**Gloeobacteria** (1)	**1/0**	**1/0**	**0/1**	**0/1**	**1/0**	**0/1**	**0/0**	**1/0**
Gloeobacter violaceus PCC 7421	C+	C+	^{4}C-	^{3}C-	C+	^{4}C-		^{h}C+
**Unknown (class)** (32)	**0/32**	**0/32**	**0/32**	**0/31**	**0/32**	**0/31**	**14/8**	**23/3**
Acaryochloris marina MBIC11017	^{4}C-	^{3}C-	^{4}C-	^{3}C-	^{4}C-	^{4}C-		
Anabaena variabilis ATCC 29413	^{4}C-	^{3}C-	^{4}C-	^{3}C-	^{4}C-	^{4}C-	C+	^{h}C+
Cyanothece sp. ATCC 51142	^{4}C-	^{3}C-	^{4}C-	^{3}C-	^{4}C-	^{4}C-	C+	^{h}C+
Microcystis aeruginosa NIES-843	^{4}C-	^{3}C-	^{4}C-	^{3}C-	^{4}C-		C+	
Nostoc punctiforme PCC 73102	^{4}C-	^{3}C-	^{4}C-		^{4}C-	^{4}C-	C+	^{h}C+
Nostoc sp. PCC 7120	^{4}C-	^{3}C-	^{4}C-	^{3}C-	^{4}C-	^{4}C-	C+	^{h}C+

Table 1: (cont) Sequences used in the study

Taxon	S4	S14	S18	L28	L31	L32	L33	L36
**Prochlorococcus marinus**								
Prochlorococcus marinus str. AS9601	^{4}C-	^{2}C-	^{4}C-	^{3}C-	^{4}C-	^{4}C-		
Prochlorococcus marinus str. MIT 9211	^{4}C-	^{2}C-	^{4}C-	^{3}C-	^{4}C-	^{4}C-	^{1}C-	^{h}C+
Prochlorococcus marinus str. MIT 9215	^{4}C-	^{2}C-	^{4}C-	^{3}C-	^{4}C-	^{4}C-		
Prochlorococcus marinus str. MIT 9301	^{4}C-	^{2}C-	^{4}C-	^{3}C-	^{4}C-	^{4}C-		
Prochlorococcus marinus str. MIT 9303	^{4}C-	^{2}C-	^{4}C-	^{3}C-	^{4}C-	^{4}C-	C+	^{h}C+
Prochlorococcus marinus str. MIT 9312	^{4}C-	^{2}C-	^{4}C-	^{3}C-	^{4}C-	^{4}C-		^{4}C-
Prochlorococcus marinus str. MIT 9313	^{4}C-	^{2}C-	^{4}C-	^{3}C-	^{4}C-	^{4}C-	C+	^{h}C+
Prochlorococcus marinus str. MIT 9515	^{4}C-	^{2}C-	^{4}C-	^{3}C-	^{4}C-	^{4}C-		^{h}C+/^{4}C-
Prochlorococcus marinus str. NATL1A	^{4}C-	^{2}C-	^{4}C-	^{3}C-	^{4}C-	^{4}C-	^{2}C-	
Prochlorococcus marinus str. NATL2A	^{4}C-	^{2}C-	^{4}C-	^{3}C-	^{4}C-	^{4}C-		^{4}C-
Prochlorococcus marinus subsp. marinus str. CCMP1375	^{4}C-	^{2}C-	^{4}C-	^{3}C-	^{4}C-	^{4}C-	^{1}C-	^{h}C+
Prochlorococcus marinus subsp. pastoris str. CCMP1986	^{4}C-	^{2}C-	^{4}C-	^{3}C-	^{4}C-	^{4}C-		^{h}C+
**Synechococcus elongatus**								
Synechococcus elongatus PCC 6301	^{4}C-	^{2}C-	^{4}C-	^{3}C-	^{4}C-	^{4}C-	C+	^{h}C+
Synechococcus elongatus PCC 7942	^{4}C-	^{2}C-	^{4}C-	^{3}C-	^{4}C-	^{4}C-	C+	^{h}C+
Synechococcus sp. CC9311	^{4}C-	^{2}C-	^{4}C-	^{3}C-	^{4}C-	^{4}C-	^{1}C-	^{h}C+
Synechococcus sp. CC9605	^{4}C-	^{2}C-	^{4}C-	^{3}C-	^{4}C-	^{4}C-	C+/^{1}C-	^{h}C+
Synechococcus sp. CC9902	^{4}C-	^{2}C-	^{4}C-	^{3}C-	^{4}C-	^{4}C-	^{1}C-	^{h}C+
Synechococcus sp. JA-2-3B'a(2-13)	^{4}C-	^{2}C-	^{4}C-	^{3}C-	^{4}C-	^{4}C-		^{h}C+
Synechococcus sp. JA-3-3Ab	^{4}C-	^{2}C-	^{4}C-	^{3}C-	^{4}C-	^{4}C-		^{h}C+
Synechococcus sp. PCC 7002	^{4}C-	^{2}C-	^{4}C-	^{3}C-	^{4}C-	^{4}C-	C+	
Synechococcus sp. RCC307	^{4}C-	^{2}C-	^{4}C-	^{3}C-	^{4}C-	^{4}C-	^{1}C-	^{h}C+
Synechococcus sp. WH 7803	^{4}C-	^{2}C-	^{4}C-	^{3}C-	^{4}C-	^{4}C-	^{1}C-	^{h}C+
Synechococcus sp. WH 8102	^{4}C-	^{2}C-	^{4}C-	^{3}C-	^{4}C-	^{4}C-	C+	^{h}C+
Synechocystis sp. PCC 6803	^{4}C-	^{2}C-	^{4}C-	^{3}C-	^{4}C-	^{4}C-	C+	^{h}C+
Thermosynechococcus elongatus BP-1	^{4}C-	^{2}C-	^{4}C-	^{3}C-	^{4}C-	^{4}C-		^{h}C+
Trichodesmium erythraeum IMS101	^{4}C-	^{3}C-	^{4}C-	^{3}C-	^{4}C-	^{4}C-	C+	^{h}C+
**Deinococcus-Thermus** (4)	**2/2**	**3/1**	**0/4**	**1/4**	**2/2**	**4/0**	**2/2**	**4/0**
**Deinococci** (4)	**2/2**	**3/1**	**0/4**	**1/4**	**2/2**	**4/0**	**2/2**	**4/0**
Deinococcus geothermalis DSM 11300	^{4}C-	C+	^{4}C-	C+/^{3}C-	^{4}C-	C+	^{4}C-	^{h}C+
Deinococcus radiodurans R1	^{4}C-	^{3}C-	^{4}C-	^{3}C-	^{4}C-	C+	^{4}C-	^{h}C+
**Thermus thermophilus**								
Thermus thermophilus HB27	C+	C+	^{4}C-	^{3}C-	C+	C+	C+	^{h}C+
Thermus thermophilus HB8	C+	C+	^{4}C-	^{3}C-	C+	C+	C+	^{h}C+
**Firmicutes** (127)	**32/112**	**114/69**	**32/95**	**31/92**	**40/90**	**77/47**	**61/137**	**112/0**
**Bacilli** (93)	**0/93**	**82/69**	**0/94**	**0/92**	**7/90**	**48/47**	**31/137**	**80/0**
Bacillus amyloliquefaciens FZB42	^{4}C-	C+/^{3}C-	^{3}C-	^{3}C-	C+/^{4}C-	C+	C+/^{1}C-/^{3}C-	^{h}C+
**Bacillus anthracis**								
Bacillus anthracis str. 'Ames Ancestor'	^{4}C-	C+	^{3}C-	^{3}C-	^{4}C-	C+	^{1}C-/^{1}C-	^{h}C+
Bacillus anthracis str. Ames	^{4}C-	C+	^{3}C-	^{3}C-	^{4}C-	C+	^{1}C-/^{1}C-	^{h}C+
Bacillus anthracis str. Sterne	^{4}C-	C+	^{3}C-	^{3}C-	^{4}C-	C+	^{1}C-/^{1}C-	^{h}C+
**Bacillus cereus**								
Bacillus cereus ATCC 10987	^{4}C-	C+	^{3}C-	^{3}C-	^{4}C-	C+	^{1}C-/^{1}C-	^{h}C+
Bacillus cereus ATCC 14579	^{4}C-	C+	^{3}C-	^{3}C-	^{4}C-		^{1}C-/^{1}C-	^{h}C+
Bacillus cereus E33L	^{4}C-	C+	^{3}C-	^{3}C-	^{4}C-	C+		

Table 1: (cont) Sequences used in the study

Taxon	S4	S14	S18	L28	L31	L32	L33	L36
Bacillus cereus subsp. cytotoxis NVH 391-98	^{4}C-	C+	^{3}C-	^{3}C-	^{4}C-	C+	^{1}C-/^{1}C-	^{h}C+
Bacillus clausii KSM-K16	^{4}C-	C+/^{3}C-	^{3}C-	^{3}C-	^{4}C-	C+	^{3}C-	^{h}C+
Bacillus halodurans C-125	^{4}C-	C+	^{3}C-	^{3}C-	^{4}C-	C+	C+	^{h}C+
**Bacillus licheniformis**								
Bacillus licheniformis ATCC 14580	^{4}C-	C+/C+/C+/C+/^{3}C-/^{3}C- /^{3}C-		^{3}C-/^{3}C-	C+/C+/^{4}C- /^{4}C-	C+/C+	C+/C+/^{1}C- /^{3}C-/^{1}C- /^{3}C-	^{h}C+/^{h}C+
Bacillus licheniformis DSM 13	^{4}C-							
Bacillus pumilus SAFR-032	^{4}C-	C+/^{3}C-	^{3}C-	^{3}C-	C+/^{4}C-	C+	C+/^{3}C- /^{1}C-	^{h}C+
Bacillus subtilis subsp. subtilis str. 168	^{4}C-	C+/^{3}C-	^{3}C-	^{3}C-	C+/^{4}C-	C+	C+/^{1}C-	^{h}C+
**Bacillus thuringiensis**								
Bacillus thuringiensis serovar konkukian str. 97-27	^{4}C-	C+	^{3}C-	^{3}C-	^{4}C-	C+		
Bacillus thuringiensis str. Al Hakam	^{4}C-	C+	^{3}C-	^{3}C-				
Bacillus weihenstephanensis KBAB4	^{4}C-	C+	^{3}C-	^{3}C-	^{4}C-	C+	^{1}C-	^{h}C+
Enterococcus faecalis V583	^{4}C-	C+/^{4}C- /^{4}C-	^{4}C-	^{3}C-	^{4}C-	C+/^{4}C-	^{1}C-/^{3}C- /^{3}C-	^{h}C+
Exiguobacterium sibiricum 255-15	^{4}C-	^{3}C-	^{3}C-	^{3}C-	^{4}C-	C+	C+/^{3}C-	^{h}C+
Geobacillus kaustophilus HTA426	^{4}C-	C+	^{3}C-	^{3}C-	C+	C+	C+/^{1}C-	^{h}C+
Geobacillus thermodenitrificans NG80-2	^{4}C-	C+	^{3}C-	^{3}C-	C+	C+	C+/^{1}C-	
Lactobacillus acidophilus NCFM	^{4}C-		^{4}C-	^{4}C-	^{4}C-	^{4}C-	^{1}C-	^{h}C+
Lactobacillus brevis ATCC 367	^{4}C-	C+/^{4}C-	^{4}C-	^{4}C-	^{4}C-	C+	C+/^{1}C-	^{h}C+
Lactobacillus casei ATCC 334	^{4}C-	C+/^{4}C- /^{4}C-	^{4}C-	^{4}C-	^{4}C-	^{4}C-	^{4}C-	^{h}C+
**Lactobacillus delbrueckii**								
Lactobacillus delbrueckii subsp. bulgaricus ATCC 11842	^{4}C-	C+	^{4}C-	^{4}C-	^{4}C-	^{4}C-	C+/^{1}C-	^{h}C+
Lactobacillus delbrueckii subsp. bulgaricus ATCC BAA-365	^{4}C-	C+	^{4}C-	^{4}C-	^{4}C-	^{4}C-	C+/^{1}C-	^{h}C+
Lactobacillus fermentum IFO 3956	^{4}C-	C+/^{4}C-	^{4}C-	^{4}C-	^{4}C-	C+	C+/^{1}C-	^{h}C+
Lactobacillus gasseri ATCC 33323	^{4}C-	^{4}C-	^{4}C-	^{4}C-	^{4}C-	^{4}C-	C+/^{1}C-	^{h}C+
Lactobacillus helveticus DPC 4571	^{4}C-	C+/C+	^{4}C-	^{4}C-	^{4}C-	^{4}C-	^{1}C-	
Lactobacillus johnsonii NCC 533	^{4}C-	C+/^{4}C-	^{4}C-	^{4}C-	^{4}C-	^{4}C-	C+/^{1}C-	^{h}C+
Lactobacillus plantarum WCFS1	^{4}C-	C+/^{4}C-	^{4}C-	^{4}C-	^{4}C-	C+	C+	^{h}C+
Lactobacillus reuteri F275	^{4}C-	C+/C+/^{4}C- /^{4}C-	^{4}C-/^{4}C-	^{4}C-/^{4}C-	^{4}C-/^{4}C-	C+/C+	^{1}C-/^{1}C-	^{h}C+
Lactobacillus sakei subsp. sakei 23K	^{4}C-	^{4}C-	^{4}C-	^{4}C-	^{4}C-	^{4}C-	C+/^{1}C-	^{h}C+
Lactobacillus salivarius UCC118	^{4}C-	C+/^{4}C-	^{4}C-	^{4}C-	^{4}C-	C+	C+/^{1}C-	^{h}C+
**Lactococcus lactis**								
Lactococcus lactis subsp. cremoris MG1363	^{4}C-	C+	^{4}C-	^{3}C-	^{4}C-	^{4}C-	C+/^{1}C- /^{4}C-	^{h}C+
Lactococcus lactis subsp. cremoris SK11	^{4}C-	C+	^{4}C-	^{3}C-	^{4}C-	^{4}C-	C+/^{1}C- /^{4}C-	^{h}C+
Lactococcus lactis subsp. lactis Il1403	^{4}C-	C+/^{4}C-	^{4}C-	^{3}C-	^{4}C-	^{4}C-	C+/^{1}C- /^{4}C-	^{h}C+
Leuconostoc citreum KM20	^{4}C-	^{4}C-	^{4}C-	^{4}C-	^{4}C-	^{4}C-	^{4}C-	
Leuconostoc mesenteroides subsp. mesenteroides ATCC 8293	^{4}C-	^{4}C-	^{4}C-	^{4}C-	^{4}C-	^{4}C-	C+/^{4}C-	^{h}C+
Listeria innocua Clip11262	^{4}C-	C+/^{3}C-	^{3}C-	^{3}C-	^{4}C-	C+/^{4}C-	C+/^{1}C-	^{h}C+
**Listeria monocytogenes**								
Listeria monocytogenes EGD-e	^{4}C-	C+/^{3}C-	^{3}C-	^{3}C-	^{4}C-	C+/^{4}C-	C+/^{1}C-	^{h}C+
Listeria monocytogenes str. 4b F2365	^{4}C-	C+/^{3}C-	^{3}C-	^{3}C-	^{4}C-	C+/^{4}C-	C+/^{1}C-	^{h}C+
Listeria welshimeri serovar 6b str. SLCC5334	^{4}C-	C+/^{3}C-	^{3}C-	^{3}C-	^{4}C-	C+/^{4}C-	C+/^{1}C-	^{h}C+
Lysinibacillus sphaericus C3-41	^{4}C-	C+	^{3}C-			C+	^{3}C-	
Oceanobacillus iheyensis HTE831	^{4}C-	C+/^{3}C-	^{3}C-	^{3}C-	^{4}C-	C+	C+/^{3}C-	^{h}C+
Oenococcus oeni PSU-1	^{4}C-	^{4}C-	^{4}C-	^{4}C-	^{4}C-	^{4}C-	^{4}C-	^{h}C+
Pediococcus pentosaceus ATCC 25745	^{4}C-	C+/^{4}C-	^{4}C-	^{4}C-	^{4}C-	C+	C+/^{1}C-	^{h}C+
**Staphylococcus aureus**								

Table 1: (cont) Sequences used in the study

Taxon	S4	S14	S18	L28	L31	L32	L33	L36
Staphylococcus aureus RF122	^{4}C-	C+/^{3}C-	^{3}C-	^{3}C-	^{4}C-	C+	^{1}C-/^{1}C-	
Staphylococcus aureus subsp. aureus COL	^{4}C-	C+/^{3}C-	^{3}C-	^{3}C-	^{4}C-	C+	^{1}C-/^{1}C-	^{h}C+
Staphylococcus aureus subsp. aureus JH1	^{4}C-	C+/^{3}C-	^{3}C-	^{3}C-	^{4}C-	C+	^{1}C-/^{1}C-	^{h}C+
Staphylococcus aureus subsp. aureus JH9	^{4}C-	C+/^{3}C-	^{3}C-	^{3}C-	^{4}C-	C+	^{1}C-/^{1}C-	^{h}C+
Staphylococcus aureus subsp. aureus MRSA252	^{4}C-	C+/^{3}C-	^{3}C-	^{3}C-	^{4}C-	C+	^{1}C-/^{1}C-	^{h}C+
Staphylococcus aureus subsp. aureus MSSA476	^{4}C-	C+/^{3}C-	^{3}C-	^{3}C-	^{4}C-	C+	^{1}C-/^{1}C-	^{h}C+
Staphylococcus aureus subsp. aureus MW2	^{4}C-	C+/^{3}C-	^{3}C-	^{3}C-	^{4}C-	C+	^{1}C-/^{1}C-	^{h}C+
Staphylococcus aureus subsp. aureus Mu3	^{4}C-	C+/^{3}C-	^{3}C-	^{3}C-	^{4}C-	C+	^{1}C-/^{1}C-	^{h}C+
Staphylococcus aureus subsp. aureus Mu50	^{4}C-	C+/^{3}C-	^{3}C-	^{3}C-	^{4}C-	C+	^{1}C-/^{1}C-	^{h}C+
Staphylococcus aureus subsp. aureus N315	^{4}C-	C+/^{3}C-	^{3}C-	^{3}C-	^{4}C-	C+	^{1}C-/^{1}C-	^{h}C+
Staphylococcus aureus subsp. aureus NCTC 8325	^{4}C-	C+/^{3}C-	^{3}C-	^{3}C-	^{4}C-	^{1}C-	^{1}C-/^{1}C-	^{h}C+
Staphylococcus aureus subsp. aureus USA300	^{4}C-	C+/^{3}C-	^{3}C-	^{3}C-	^{4}C-	C+	^{1}C-/^{1}C-	^{h}C+
Staphylococcus aureus subsp. aureus USA300 TCH1516	^{4}C-	C+	^{3}C-		^{4}C-		^{1}C-/^{1}C-	^{h}C+
Staphylococcus aureus subsp. aureus str. Newman	^{4}C-	C+/^{3}C-	^{3}C-	^{3}C-	^{4}C-	C+	^{1}C-/^{1}C-	^{h}C+
**Staphylococcus epidermidis**								
Staphylococcus epidermidis ATCC 12228	^{4}C-	C+/^{3}C-	^{3}C-	^{3}C-	^{4}C-	C+	^{1}C-/^{1}C-	^{h}C+
Staphylococcus epidermidis RP62A	^{4}C-	C+/^{3}C-	^{3}C-	^{3}C-	^{4}C-	C+	^{1}C-/^{1}C-	^{h}C+
Staphylococcus haemolyticus JCSC1435	^{4}C-	C+/^{3}C-	^{3}C-	^{3}C-	^{4}C-	C+	^{1}C-/^{1}C-	^{h}C+
Staphylococcus saprophyticus subsp. saprophyticus ATCC 15305	^{4}C-	C+/^{3}C-	^{3}C-	^{3}C-	^{4}C-	C+	C+/^{1}C-/^{1}C-	^{h}C+
**Streptococcus agalactiae**								
Streptococcus agalactiae 2603V/R	^{4}C-	C+/^{4}C-	^{4}C-	^{3}C-	^{4}C-	^{4}C-	C+/^{1}C-/^{4}C-	^{h}C+
Streptococcus agalactiae A909	^{4}C-	C+/^{4}C-	^{4}C-	^{3}C-	^{4}C-	^{4}C-	^{4}C-	
Streptococcus agalactiae NEM316	^{4}C-	C+/^{4}C-	^{4}C-	^{3}C-	^{4}C-	^{4}C-	^{4}C-	^{h}C+
Streptococcus gordonii str. Challis substr. CH1	^{4}C-	C+	^{4}C-	^{3}C-	^{4}C-	^{4}C-	^{4}C-	^{h}C+
Streptococcus mutans UA159	^{4}C-	C+	^{4}C-	^{3}C-	^{4}C-	^{4}C-	^{1}C-	^{h}C+
**Streptococcus pneumoniae**								
Streptococcus pneumoniae CGSP14	^{4}C-	^{3}C-	^{4}C-	^{3}C-	^{4}C-	^{4}C-	^{4}C-	
Streptococcus pneumoniae D39	^{4}C-	^{3}C-	^{4}C-	^{3}C-	^{4}C-	^{4}C-	^{4}C-	
Streptococcus pneumoniae Hungary19A-6	^{4}C-	^{3}C-	^{4}C-	^{3}C-	^{4}C-	^{4}C-	^{1}C-/^{4}C-	^{h}C+
Streptococcus pneumoniae R6	^{4}C-	^{3}C-	^{4}C-	^{3}C-	^{4}C-	^{4}C-	^{1}C-/^{4}C-	^{h}C+
Streptococcus pneumoniae TIGR4	^{4}C-	^{3}C-	^{4}C-	^{3}C-	^{4}C-	^{4}C-	^{1}C-/^{4}C-	^{h}C+
**Streptococcus pyogenes**								
Streptococcus pyogenes M1 GAS	^{4}C-	C+/^{4}C-	^{4}C-	^{3}C-	^{4}C-		^{4}C-	^{h}C+
Streptococcus pyogenes MGAS10270	^{4}C-	C+/^{4}C-	^{4}C-	^{3}C-	^{4}C-	^{4}C-	^{1}C-/^{4}C-	^{h}C+
Streptococcus pyogenes MGAS10394	^{4}C-	C+/^{4}C-	^{4}C-	^{3}C-	^{4}C-	^{4}C-	^{1}C-/^{4}C-	^{h}C+
Streptococcus pyogenes MGAS10750	^{4}C-	C+/^{4}C-	^{4}C-	^{3}C-	^{4}C-	^{4}C-	^{1}C-/^{4}C-	^{h}C+
Streptococcus pyogenes MGAS2096	^{4}C-	^{4}C-	^{4}C-	^{3}C-	^{4}C-	^{4}C-	^{1}C-/^{4}C-	^{h}C+
Streptococcus pyogenes MGAS315	^{4}C-	C+/^{4}C-	^{4}C-	^{3}C-	^{4}C-	^{4}C-	^{4}C-	^{h}C+
Streptococcus pyogenes MGAS5005	^{4}C-	C+/^{4}C-	^{4}C-	^{3}C-	^{4}C-	^{4}C-	^{1}C-/^{4}C-	^{h}C+
Streptococcus pyogenes MGAS6180	^{4}C-	C+/^{4}C-	^{4}C-	^{3}C-	^{4}C-	^{4}C-	^{1}C-/^{4}C-	^{h}C+
Streptococcus pyogenes MGAS8232	^{4}C-	C+/^{4}C-	^{4}C-	^{3}C-	^{4}C-	^{4}C-	^{4}C-	^{h}C+
Streptococcus pyogenes MGAS9429	^{4}C-	C+/^{4}C-	^{4}C-	^{3}C-	^{4}C-	^{4}C-	^{1}C-/^{4}C-	^{h}C+
Streptococcus pyogenes SSI-1	^{4}C-	C+/^{4}C-	^{4}C-	^{3}C-	^{4}C-	^{4}C-	^{1}C-/^{4}C-	^{h}C+
Streptococcus pyogenes str. Manfredo	^{4}C-	C+/^{4}C-	^{4}C-	^{3}C-	^{4}C-	^{4}C-	^{1}C-/^{4}C-	^{h}C+
Streptococcus sanguinis SK36	^{4}C-	C+	^{4}C-	^{3}C-	^{4}C-	^{4}C-		^{h}C+
**Streptococcus suis**								

Table 1: (cont) Sequences used in the study

Taxon	S4	S14	S18	L28	L31	L32	L33	L36
Streptococcus suis 05ZYH33	^{4}C-	^{4}C-	^{4}C-	^{3}C-	^{4}C-	^{4}C-	^{4}C-	
Streptococcus suis 98HAH33	^{4}C-	^{4}C-	^{4}C-	^{3}C-	^{4}C-	^{4}C-	^{4}C-	
**Streptococcus thermophilus**								
Streptococcus thermophilus CNRZ1066	^{4}C-	C+	^{4}C-	^{3}C-	^{4}C-	^{4}C-	^{1}C-/^{4}C-	^{h}C+
Streptococcus thermophilus LMD-9	^{4}C-	C+	^{4}C-	^{3}C-	^{4}C-	^{4}C-	C+/^{1}C-/^{4}C-	^{h}C+
Streptococcus thermophilus LMG 18311	^{4}C-	C+	^{4}C-	^{3}C-	^{4}C-	^{4}C-	^{1}C-/^{4}C-	^{h}C+
**Clostridia** (34)	**32/19**	**32/0**	**32/1**	**31/0**	**33/0**	**29/0**	**30/0**	**32/0**
Alkaliphilus metalliredigens QYMF	C+/^{4}C-/^{3}C-	C+	^{h}C+	C+	C+	C+	C+	^{h}C+
Alkaliphilus oremlandii OhILAs	C+/^{4}C-	C+	^{h}C+	C+	C+	C+	C+	^{h}C+
Caldicellulosiruptor saccharolyticus DSM 8903	C+	C+	^{h}C+	C+	C+	C+	C+	^{h}C+
Candidatus Desulforudis audaxviator MP104C	C+	C+	^{h}C+	C+	C+	C+	C+	^{h}C+
Carboxydothermus hydrogenoformans Z-2901	C+	C+	^{h}C+	C+	C+	C+	C+	^{h}C+
Clostridium acetobutylicum ATCC 824	C+/^{4}C-/^{4}C-	C+	^{h}C+	C+	C+	C+	C+	^{h}C+
Clostridium beijerinckii NCIMB 8052	C+	C+	^{h}C+	C+	C+	C+	C+	^{h}C+
**Clostridium botulinum**								
Clostridium botulinum A str. ATCC 19397	C+/^{4}C-	C+	^{h}C+	C+	C+	C+	C+	^{h}C+
Clostridium botulinum A str. ATCC 3502	C+/^{4}C-	C+	^{h}C+	C+	C+	C+	C+	^{h}C+
Clostridium botulinum A str. Hall	C+/^{4}C-	C+	^{h}C+	C+	C+	C+	C+	^{h}C+
Clostridium botulinum A3 str. Loch Maree	C+/^{4}C-	C+	^{h}C+	C+	C+	C+	C+	^{h}C+
Clostridium botulinum B str. Eklund 17B	C+	C+	^{h}C+	C+	C+	C+		^{h}C+
Clostridium botulinum B1 str. Okra	C+/^{4}C-	C+	^{h}C+	C+	C+	C+	C+	^{h}C+
Clostridium botulinum F str. Langeland	C+/^{4}C-	C+	^{h}C+	C+	C+	C+	C+	^{h}C+
Clostridium difficile 630	C+	C+	^{h}C+	C+	C+	C+	C+	^{h}C+
Clostridium kluyveri DSM 555	C+/^{3}C-	C+	^{h}C+	C+	C+		C+	^{h}C+
Clostridium novyi NT	C+/^{4}C-	C+	^{h}C+	C+	C+	C+		^{h}C+
**Clostridium perfringens**								
Clostridium perfringens ATCC 13124	C+/^{3}C-	C+	^{h}C+	C+	C+	C+	C+	^{h}C+
Clostridium perfringens SM101	C+/^{3}C-	C+	^{h}C+	C+	C+	C+	C+	^{h}C+
Clostridium perfringens str. 13	C+/^{3}C-	C+	^{h}C+	C+	C+	C+	C+	^{h}C+
Clostridium phytofermentans ISDg	^{3}C-	C+	^{h}C+		C+	C+	C+	^{h}C+
Clostridium tetani E88	C+							
Clostridium thermocellum ATCC 27405	C+	C+	^{h}C+	C+	C+	C+	C+	^{h}C+
Desulfitobacterium hafniense Y51	C+	C+	^{h}C+	C+	C+	C+	C+	^{h}C+
Desulfotomaculum reducens MI-1	C+	C+	^{h}C+	C+	C+	C+	C+	^{h}C+
Finegoldia magna ATCC 29328	^{4}C-	C+	^{4}C-	C+	C+	C+	C+	
Heliobacterium modesticaldum Ice1	C+	C+	^{h}C+	C+	C+	C+	C+	^{h}C+
Moorella thermoacetica ATCC 39073	C+	C+	^{h}C+	C+	C+		C+	^{h}C+
Pelotomaculum thermopropionicum SI	C+		^{h}C+		C+		C+	^{h}C+
Symbiobacterium thermophilum IAM 14863	C+/^{3}C-	C+	^{h}C+	C+	C+	C+	C+	^{h}C+
Syntrophomonas wolfei subsp. wolfei str. Goettingen	C+	C+	^{h}C+	C+	C+	C+	C+	^{h}C+
Thermoanaerobacter pseudethanolicus ATCC 33223	C+	C+	^{h}C+	C+	C+	C+	C+	^{h}C+
Thermoanaerobacter sp. X514	C+	C+	^{h}C+	C+	C+	C+		^{h}C+
Thermoanaerobacter tengcongensis MB4	C+	C+	^{h}C+	C+	C+		C+	^{h}C+
**Fusobacteria** (6)	**0/6**	**0/1**	**0/1**	**0/1**	**0/1**	**1/0**	**0/1**	**0/0**
**Fusobacteria (class)** (6)	**0/6**	**0/1**	**0/1**	**0/1**	**0/1**	**1/0**	**0/1**	**0/0**
**Fusobacterium nucleatum**								

Table 1: (cont) Sequences used in the study

Taxon	S4	S14	S18	L28	L31	L32	L33	L36
Fusobacterium nucleatum subsp. nucleatum ATCC 25586	^{3}C-	^{3}C-	^{4}C-	^{2}C-	^{4}C-	C+	^{3}C-	
Fusobacterium nucleatum subsp. polymorphum ATCC 10953 *	^{3}C-							
Fusobacterium nucleatum subsp. vincentii ATCC 49256 *	^{3}C-							
Leptotrichia buccalis DSM 1135 *	^{3}C-							
Sebaldella termitidis ATCC 33386 *	^{3}C-							
Streptobacillus moniliformis DSM 12112 *	^{3}C-							
**Planctomycetes** (4)	**1/4**	**1/0**	**0/1**	**0/1**	**1/0**	**0/0**	**0/1**	**0/1**
**Planctomycetacia** (4)	**1/4**	**1/0**	**0/1**	**0/1**	**1/0**	**0/0**	**0/1**	**0/1**
Blastopirellula marina DSM 3645 *	^{4}C-							
Gemmata obscuriglobus UQM 2246 *	C+/^{4}C-							
Planctomyces maris DSM 8797 *	^{4}C-							
Rhodopirellula baltica SH 1	^{4}C-	C+	^{4}C-	^{4}C-	C+		^{3}C-	^{2}C-
**Proteobacteria** (347)	**27/325**	**41/308**	**21/329**	**16/325**	**186/234**	**31/260**	**14/310**	**188/152**
**Alphaproteobacteria** (87)	**0/87**	**0/89**	**0/89**	**0/86**	**0/89**	**18/67**	**0/74**	**0/84**
Acidiphilium cryptum JF-5	^{4}C-	^{2}C-	^{3}C-	^{3}C-	^{4}C-	C+	^{4}C-	^{3}C-
Agrobacterium tumefaciens str. C58	^{4}C-	^{3}C-	^{3}C-	^{3}C-	^{4}C-	^{4}C-	^{4}C-	^{4}C-
Anaplasma marginale str. St. Maries	^{4}C-	^{3}C-	^{3}C-	^{3}C-	^{4}C-	^{3}C-	^{4}C-	^{3}C-
Anaplasma phagocytophilum HZ	^{4}C-	^{3}C-	^{3}C-	^{3}C-	^{4}C-	^{3}C-	^{4}C-	^{3}C-
Azorhizobium caulinodans ORS 571	^{4}C-	^{3}C-	^{3}C-	^{3}C-	^{4}C-	^{4}C-	^{4}C-	^{4}C-
Bartonella bacilliformis KC583	^{4}C-	^{3}C-	^{3}C-	^{3}C-	^{4}C-	^{4}C-	^{4}C-	^{4}C-
Bartonella henselae str. Houston-1	^{4}C-	^{3}C-	^{3}C-	^{3}C-	^{4}C-	^{4}C-	^{4}C-	^{4}C-
Bartonella quintana str. Toulouse	^{4}C-	^{3}C-	^{3}C-	^{3}C-	^{4}C-	^{4}C-	^{4}C-	^{4}C-
Bartonella tribocorum CIP 105476	^{4}C-	^{3}C-	^{3}C-	^{3}C-	^{4}C-	^{4}C-	^{4}C-	^{4}C-
Beijerinckia indica subsp. indica ATCC 9039	^{4}C-	^{3}C-	^{3}C-	^{3}C-	^{4}C-	^{4}C-	^{4}C-	^{4}C-
Bradyrhizobium japonicum USDA 110	^{4}C-	^{3}C-	^{3}C-	^{3}C-	^{4}C-	^{4}C-	^{4}C-	^{4}C-
Bradyrhizobium sp. BTAi1	^{4}C-	^{3}C-	^{3}C-	^{3}C-	^{4}C-		^{4}C-	^{4}C-
Bradyrhizobium sp. ORS278	^{4}C-	^{3}C-	^{3}C-	^{3}C-	^{4}C-	^{4}C-	^{4}C-	^{4}C-
**Brucella abortus**								
Brucella abortus S19	^{4}C-	^{3}C-	^{3}C-	^{3}C-	^{4}C-	^{4}C-	^{4}C-	^{3}C-
Brucella abortus bv. 1 str. 9-941	^{4}C-	^{3}C-	^{3}C-	^{3}C-	^{4}C-	^{4}C-	^{4}C-	^{3}C-
Brucella melitensis biovar Abortus 2308	^{4}C-	^{3}C-	^{3}C-	^{3}C-	^{4}C-	^{4}C-	^{4}C-	^{3}C-
Brucella canis ATCC 23365	^{4}C-	^{3}C-	^{3}C-	^{3}C-	^{4}C-	^{4}C-	^{4}C-	^{3}C-
Brucella melitensis 16M	^{4}C-	^{3}C-	^{3}C-	^{3}C-	^{4}C-	^{4}C-	^{4}C-	^{3}C-
Brucella ovis ATCC 25840	^{4}C-	^{3}C-	^{3}C-	^{3}C-	^{4}C-	^{4}C-	^{4}C-	^{3}C-
**Brucella suis**								
Brucella suis 1330	^{4}C-	^{3}C-	^{3}C-	^{3}C-	^{4}C-	^{4}C-	^{4}C-	^{3}C-
Brucella suis ATCC 23445	^{4}C-	^{3}C-	^{3}C-	^{3}C-	^{4}C-	^{4}C-	^{4}C-	^{3}C-
Candidatus Pelagibacter ubique HTCC1062	^{4}C-	^{3}C-	^{3}C-	^{3}C-	^{4}C-	^{4}C-		^{4}C-
Caulobacter sp. K31	^{4}C-	^{3}C-	^{3}C-	^{3}C-	^{4}C-		^{4}C-	^{3}C-
Caulobacter crescentus CB15	^{4}C-	^{3}C-	^{3}C-	^{3}C-	^{4}C-	^{4}C-	^{4}C-	^{3}C-
Dinoroseobacter shibae DFL 12	^{4}C-	^{3}C-	^{3}C-	^{3}C-	^{4}C-	C+	^{4}C-	^{3}C-
Ehrlichia canis str. Jake	^{4}C-	^{3}C-	^{3}C-	^{3}C-	^{4}C-	^{3}C-	^{4}C-	^{3}C-
Ehrlichia chaffeensis str. Arkansas	^{4}C-	^{3}C-	^{3}C-	^{3}C-	^{4}C-	^{3}C-		^{3}C-
**Ehrlichia ruminantium**								
Ehrlichia ruminantium str. Gardel	^{4}C-	^{3}C-	^{3}C-	^{3}C-	^{4}C-	^{3}C-	^{4}C-	^{3}C-
Ehrlichia ruminantium str. Welgevonden	^{4}C-	^{3}C-/^{3}C-/^{3}C-/^{3}C-	^{3}C-/^{3}C-/^{3}C-/^{3}C-	^{3}C-/^{3}C-/^{3}C-/^{3}C-	^{4}C-/^{4}C-/^{4}C-/^{4}C-	^{3}C-/^{3}C-/^{3}C-/^{3}C-	^{4}C-/^{4}C-/^{4}C-/^{4}C-	^{3}C-/^{3}C-

Table 1: (cont) Sequences used in the study

Taxon	S4	S14	S18	L28	L31	L32	L33	L36
Ehrlichia ruminantium str. Welgevonden CIRAD	^{4}C-							
Erythrobacter litoralis HTCC2594	^{4}C-	^{3}C-	^{3}C-	^{3}C-	^{4}C-	C+	^{4}C-	^{3}C-
Gluconacetobacter diazotrophicus PAl 5	^{4}C-	^{2}C-	^{3}C-	^{3}C-	^{4}C-		^{4}C-	^{3}C-
Gluconobacter oxydans 621H	^{4}C-	^{3}C-	^{3}C-	^{3}C-	^{4}C-	C+	^{4}C-	^{3}C-
Granulibacter bethesdensis CGDNIH1	^{4}C-	^{2}C-	^{3}C-	^{3}C-	^{4}C-	C+	^{4}C-	^{3}C-
Hyphomonas neptunium ATCC 15444	^{4}C-	^{3}C-	^{4}C-	^{3}C-	^{4}C-	^{4}C-	^{4}C-	^{3}C-
Jannaschia sp. CCS1	^{4}C-	^{3}C-	^{4}C-	^{3}C-	^{4}C-	C+	^{4}C-	^{3}C-
Magnetospirillum magneticum AMB-1	^{4}C-	^{2}C-	^{3}C-		^{4}C-	C+	^{4}C-	^{3}C-
Maricaulis maris MCS10	^{4}C-	^{3}C-	^{3}C-	^{3}C-	^{4}C-	^{4}C-	^{4}C-	^{3}C-
Mesorhizobium loti MAFF303099	^{4}C-	^{3}C-	^{3}C-	^{3}C-	^{4}C-	^{4}C-	^{4}C-	^{4}C-
Mesorhizobium sp. BNC1	^{4}C-	^{3}C-	^{3}C-	^{3}C-	^{4}C-	^{4}C-	^{4}C-	^{4}C-
Methylobacterium extorquens PA1	^{4}C-	^{3}C-	^{3}C-	^{3}C-	^{4}C-	^{4}C-	^{4}C-	^{4}C-
Methylobacterium radiotolerans JCM 2831	^{4}C-	^{3}C-	^{3}C-	^{3}C-	^{4}C-	^{4}C-	^{4}C-	^{4}C-
Methylobacterium sp. 4-46	^{4}C-	^{3}C-	^{3}C-	^{3}C-	^{4}C-	^{4}C-	^{4}C-	^{4}C-
Neorickettsia sennetsu str. Miyayama	^{4}C-	^{3}C-	^{4}C-	^{3}C-	^{4}C-	^{4}C-	^{4}C-	
Nitrobacter hamburgensis X14	^{4}C-	^{3}C-	^{3}C-	^{3}C-	^{4}C-	^{4}C-	^{4}C-	^{4}C-
Nitrobacter winogradskyi Nb-255	^{4}C-	^{3}C-	^{3}C-	^{3}C-	^{4}C-	^{4}C-	^{4}C-	^{4}C-
Novosphingobium aromaticivorans DSM 12444	^{4}C-	^{2}C-	^{3}C-	^{3}C-	^{4}C-	C+	^{4}C-	^{4}C-
Ochrobactrum anthropi ATCC 49188	^{4}C-	^{3}C-	^{3}C-	^{3}C-	^{4}C-	^{4}C-	^{4}C-	^{3}C-
**Orientia tsutsugamushi**								
Orientia tsutsugamushi str. Boryong	^{4}C-	^{2}C-	^{2}C-	^{3}C-	^{4}C-	^{3}C-	^{4}C-	^{3}C-
Orientia tsutsugamushi str. Ikeda	^{4}C-	^{2}C-	^{2}C-	^{3}C-	^{4}C-	^{3}C-		^{3}C-
Paracoccus denitrificans PD1222	^{4}C-	^{3}C-	^{3}C-	^{3}C-	^{4}C-	C+	^{4}C-	^{3}C-
Parvibaculum lavamentivorans DS-1	^{4}C-	^{3}C-	^{3}C-	^{3}C-	^{4}C-	^{4}C-	^{4}C-	^{4}C-
**Rhizobium etli**								
Rhizobium etli CFN 42	^{4}C-	^{3}C-	^{3}C-	^{3}C-	^{4}C-	^{4}C-		^{4}C-
Rhizobium etli CIAT 652	^{4}C-	^{3}C-	^{3}C-	^{3}C-	^{4}C-	^{4}C-		^{4}C-
Rhizobium leguminosarum bv. viciae 3841	^{4}C-	^{3}C-	^{3}C-		^{4}C-	^{4}C-	^{4}C-	
**Rhodobacter sphaeroides**								
Rhodobacter sphaeroides 2.4.1	^{4}C-	^{3}C-	^{3}C-	^{3}C-	^{4}C-	C+	^{4}C-	^{3}C-
Rhodobacter sphaeroides ATCC 17025	^{4}C-	^{3}C-	^{3}C-	^{3}C-	^{4}C-	C+	^{4}C-	^{3}C-
Rhodobacter sphaeroides ATCC 17029	^{4}C-	^{3}C-	^{3}C-	^{3}C-	^{4}C-	C+	^{4}C-	^{3}C-
**Rhodopseudomonas palustris**								
Rhodopseudomonas palustris BisA53	^{4}C-	^{3}C-	^{3}C-	^{3}C-	^{4}C-	^{4}C-	^{4}C-	^{4}C-
Rhodopseudomonas palustris BisB18	^{4}C-	^{3}C-	^{3}C-	^{3}C-	^{4}C-	^{4}C-	^{4}C-	^{4}C-
Rhodopseudomonas palustris BisB5	^{4}C-	^{3}C-	^{3}C-	^{3}C-	^{4}C-	^{4}C-	^{4}C-	^{4}C-
Rhodopseudomonas palustris CGA009	^{4}C-	^{3}C-	^{3}C-	^{3}C-	^{4}C-	^{4}C-	^{4}C-	^{4}C-
Rhodopseudomonas palustris HaA2	^{4}C-	^{3}C-	^{3}C-	^{3}C-	^{4}C-	^{4}C-	^{4}C-	^{4}C-
Rhodospirillum rubrum ATCC 11170	^{4}C-	^{3}C-	^{3}C-	^{3}C-	^{4}C-	C+	^{4}C-	^{3}C-
Rickettsia akari str. Hartford	^{4}C-	^{3}C-	^{2}C-	^{2}C-	^{4}C-	^{4}C-		^{3}C-
**Rickettsia bellii**								
Rickettsia bellii OSU 85-389	^{4}C-	^{3}C-	^{2}C-	^{2}C-	^{4}C-	^{4}C-	^{4}C-	^{3}C-
Rickettsia bellii RML369-C	^{4}C-	^{3}C-	^{2}C-	^{2}C-	^{4}C-	^{4}C-	^{4}C-	^{3}C-
Rickettsia canadensis str. McKiel	^{4}C-	^{3}C-	^{2}C-	^{2}C-	^{4}C-	^{4}C-	^{4}C-	^{3}C-
Rickettsia conorii str. Malish 7	^{4}C-	^{3}C-	^{2}C-	^{2}C-	^{4}C-	^{4}C-		^{3}C-
Rickettsia felis URRWXCal2	^{4}C-	^{3}C-	^{2}C-	^{2}C-	^{4}C-	^{4}C-		^{3}C-

Table 1: (cont) Sequences used in the study

Taxon	S4	S14	S18	L28	L31	L32	L33	L36
Rickettsia massiliae MTU5	^{4}C-	^{3}C-	^{2}C-	^{2}C-	^{4}C-	^{4}C-		^{3}C-
Rickettsia prowazekii str. Madrid E	^{4}C-	^{3}C-	^{2}C-	^{2}C-	^{4}C-	^{4}C-	^{4}C-	^{3}C-
**Rickettsia rickettsii**								
Rickettsia rickettsii str. 'Sheila Smith'	^{4}C-	^{3}C-	^{2}C-	^{2}C-	^{4}C-	^{4}C-		^{3}C-
Rickettsia rickettsii str. Iowa	^{4}C-	^{3}C-	^{2}C-	^{2}C-	^{4}C-	^{4}C-		^{3}C-
Rickettsia typhi str. Wilmington	^{4}C-	^{3}C-	^{2}C-	^{2}C-	^{4}C-	^{4}C-		^{3}C-
Roseobacter denitrificans OCh 114	^{4}C-	^{3}C-	^{3}C-	^{3}C-	^{4}C-	C+	^{4}C-	
Silicibacter pomeroyi DSS-3	^{4}C-	^{3}C-	^{3}C-	^{3}C-	^{4}C-	C+	^{4}C-	^{3}C-
Silicibacter sp. TM1040	^{4}C-	^{3}C-	^{3}C-	^{3}C-	^{4}C-	C+	^{4}C-	^{3}C-
Sinorhizobium medicae WSM419	^{4}C-	^{3}C-	^{3}C-	^{3}C-	^{4}C-	^{4}C-	^{4}C-	^{4}C-
Sinorhizobium meliloti 1021	^{4}C-	^{3}C-	^{3}C-	^{3}C-	^{4}C-	^{4}C-	^{4}C-	^{4}C-
Sphingomonas wittichii RW1	^{4}C-	^{2}C-	^{3}C-	^{3}C-	^{4}C-	C+	^{4}C-	^{4}C-
Sphingopyxis alaskensis RB2256	^{4}C-	^{2}C-	^{3}C-	^{3}C-	^{4}C-	C+	^{4}C-	^{4}C-
Wolbachia endosymbiont strain TRS of Brugia malayi	^{4}C-	^{2}C-	^{2}C-	^{3}C-	^{4}C-	^{3}C-		^{2}C-
Wolbachia endosymbiont of Drosophila melanogaster	^{4}C-	^{2}C-	^{2}C-	^{3}C-	^{4}C-	^{3}C-		^{2}C-
Wolbachia pipientis	^{4}C-	^{2}C-	^{2}C-	^{3}C-	^{4}C-	^{3}C-		^{2}C-
Xanthobacter autotrophicus Py2	^{4}C-	^{3}C-	^{3}C-	^{3}C-	^{4}C-	^{4}C-	^{4}C-	^{4}C-
Zymomonas mobilis subsp. mobilis ZM4	^{4}C-	^{2}C-	^{3}C-		^{4}C-		^{4}C-	^{4}C-
**Betaproteobacteria** (55)	**7/50**	**0/56**	**0/57**	**0/56**	**12/48**	**0/56**	**0/56**	**46/7**
Acidovorax avenae subsp. citrulli AAC00-1	C-(I)	^{3}C-	^{3}C-	^{3}C-	^{4}C-	^{4}C-	^{4}C-	^{h}C+
Acidovorax sp. JS42	C-(I)	^{3}C-	^{3}C-	^{3}C-	^{4}C-	^{4}C-	^{4}C-	^{h}C+
Azoarcus sp. BH72	C+	^{2}C-	^{3}C-	^{3}C-	C+	^{4}C-	^{4}C-	^{h}C+
Azoarcus sp. EbN1	C+	^{2}C-	^{3}C-	^{3}C-	C+	^{4}C-	^{4}C-	^{h}C+
Bordetella avium 197N	C-(I)	^{3}C-	^{3}C-	^{3}C-	^{4}C-	^{4}C-	^{4}C-	^{h}C+
Bordetella bronchiseptica RB50	C-(I)	^{3}C-	^{3}C-	^{3}C-	^{4}C-	^{4}C-	^{4}C-	^{h}C+
Bordetella parapertussis 12822	C-(I)	^{3}C-	^{3}C-	^{3}C-	^{4}C-	^{4}C-	^{4}C-	^{h}C+
Bordetella pertussis Tohama I	C-(I)	^{3}C-	^{3}C-	^{3}C-	^{4}C-	^{4}C-	^{4}C-	^{h}C+
Bordetella petrii DSM 12804	C-(I)	^{3}C-	^{3}C-	^{3}C-	^{4}C-	^{4}C-	^{4}C-	^{h}C+
**Burkholderia ambifaria**								
Burkholderia ambifaria AMMD	C-(I)	^{3}C-	^{3}C-	^{3}C-	^{4}C-	^{4}C-	^{4}C-	^{h}C+
Burkholderia ambifaria MC40-6	C-(I)	^{3}C-	^{3}C-	^{3}C-	^{4}C-	^{4}C-	^{4}C-	^{h}C+
**Burkholderia cenocepacia**								
Burkholderia cenocepacia AU 1054	C-(I)	^{3}C-	^{3}C-	^{3}C-	^{4}C-	^{4}C-	^{4}C-	^{h}C+
Burkholderia cenocepacia HI2424	C-(I)	^{3}C-	^{3}C-	^{3}C-	^{4}C-	^{4}C-	^{4}C-	^{h}C+
Burkholderia cenocepacia MC0-3	C-(I)	^{3}C-	^{3}C-	^{3}C-	^{4}C-	^{4}C-	^{4}C-	^{h}C+
**Burkholderia mallei**								
Burkholderia mallei ATCC 23344	C-(I)	^{3}C-	^{3}C-	^{3}C-	^{4}C-	^{4}C-	^{4}C-	^{h}C+
Burkholderia mallei NCTC 10229	C-(I)	^{3}C-	^{3}C-	^{3}C-	^{4}C-	^{4}C-	^{4}C-	^{h}C+
Burkholderia mallei NCTC 10247	C-(I)	^{3}C-	^{3}C-	^{4}C-	^{4}C-	^{4}C-	^{4}C-	^{h}C+
Burkholderia mallei SAVP1	C-(I)	^{3}C-	^{3}C-	^{3}C-	^{4}C-	^{4}C-	^{4}C-	^{h}C+
Burkholderia multivorans ATCC 17616	C-(I)	^{3}C-/^{3}C-	^{3}C-/^{3}C-	^{3}C-/^{3}C-	^{4}C-/^{4}C-	^{4}C-/^{4}C-	^{4}C-/^{4}C-	^{h}C+
Burkholderia phymatum STM815	C-(I)	^{3}C-	^{3}C-	^{3}C-	^{4}C-	^{4}C-	^{4}C-	^{h}C+
**Burkholderia pseudomallei**								
Burkholderia pseudomallei 1106a	C-(I)	^{3}C-	^{3}C-	^{3}C-	^{4}C-	^{4}C-	^{4}C-	^{h}C+
Burkholderia pseudomallei 1710b	C-(I)	^{3}C-	^{3}C-	^{3}C-	^{4}C-	^{4}C-	^{4}C-	^{h}C+
Burkholderia pseudomallei 668	C-(I)	^{3}C-	^{3}C-	^{3}C-	^{4}C-	^{4}C-	^{4}C-	^{h}C+

Table 1: (cont) Sequences used in the study

Taxon	S4	S14	S18	L28	L31	L32	L33	L36
Burkholderia pseudomallei K96243	C-(I)	^{3}C-	^{3}C-	^{3}C-	^{4}C-	^{4}C-	^{4}C-	^{h}C+
Burkholderia sp. 383	C-(I)	^{3}C-	^{3}C-	^{3}C-	^{4}C-	^{4}C-	^{4}C-	^{h}C+
Burkholderia thailandensis E264	C-(I)	^{3}C-	^{3}C-	^{3}C-	^{4}C-	^{4}C-	^{4}C-	^{h}C+
Burkholderia vietnamiensis G4	C-(I)	^{3}C-	^{3}C-	^{3}C-	^{4}C-	^{4}C-	^{4}C-	^{h}C+
Burkholderia xenovorans LB400	C-(I)	^{3}C-	^{3}C-	^{3}C-	^{4}C-	^{4}C-	^{4}C-	^{h}C+
Chromobacterium violaceum ATCC 12472	C-(I)	^{3}C-	^{3}C-	^{3}C-	C+	^{4}C-	^{4}C-	^{h}C+
Ralstonia metallidurans CH34	C-(I)	^{3}C-	^{3}C-	^{3}C-	^{4}C-	^{4}C-	^{4}C-	^{h}C+
**Cupriavidus necator**								
Ralstonia eutropha H16	C-(I)	^{3}C-	^{3}C-	^{3}C-	^{4}C-	^{4}C-	^{4}C-	^{h}C+
Ralstonia eutropha JMP134	C-(I)	^{3}C-	^{3}C-/^{3}C-	^{3}C-	^{3}C-	^{4}C-	^{4}C-	^{h}C+
Dechloromonas aromatica RCB	C+	^{2}C-	^{3}C-	^{3}C-	C+	^{4}C-	^{4}C-	^{h}C+
Delftia acidovorans SPH-1	C-(I)	^{3}C-	^{3}C-	^{3}C-	^{4}C-	^{4}C-	^{4}C-	^{2}C-
Herminiimonas arsenicoxydans	C-(I)	^{3}C-	^{3}C-	^{3}C-	^{4}C-	^{4}C-	^{4}C-	
Janthinobacterium sp. Marseille	C-(I)	^{3}C-	^{3}C-	^{3}C-	^{4}C-	^{4}C-	^{4}C-	^{h}C+
Leptothrix cholodnii SP-6	C-(I)	^{3}C-	^{3}C-	^{3}C-	^{4}C-	^{4}C-	^{4}C-	^{h}C+
Methylibium petroleiphilum PM1	C-(I)	^{3}C-	^{3}C-	^{3}C-	^{4}C-	^{4}C-	^{4}C-	^{h}C+
Methylobacillus flagellatus KT	^{4}C-/^{4}C-	^{3}C-	^{3}C-	^{3}C-	^{4}C-	^{4}C-	^{4}C-	^{h}C+/^{2}C-
Neisseria gonorrhoeae FA 1090	C-(I)	^{3}C-	^{3}C-	^{3}C-	C+/^{4}C-	^{4}C-	^{4}C-	^{2}C-
**Neisseria meningitidis**								
Neisseria meningitidis 053442	C-(I)	^{3}C-	^{3}C-	^{3}C-	C+/^{4}C-	^{4}C-	^{4}C-	^{h}C+/^{2}C-
Neisseria meningitidis FAM18	C-(I)	^{3}C-	^{3}C-	^{3}C-	C+/^{4}C-	^{4}C-	^{4}C-	^{h}C+/^{2}C-
Neisseria meningitidis MC58	C-(I)	^{3}C-	^{3}C-	^{3}C-	C+/^{4}C-	^{4}C-	^{4}C-	^{h}C+/^{2}C-
Neisseria meningitidis Z2491	C-(I)	^{3}C-	^{3}C-	^{3}C-	C+/^{4}C-	^{4}C-	^{4}C-	^{h}C+/^{2}C-
Nitrosomonas europaea ATCC 19718	C+/^{4}C-	^{3}C-	^{3}C-	^{3}C-	C+	^{4}C-	^{4}C-	
Nitrosomonas eutropha C91	C+	^{3}C-	^{3}C-	^{3}C-		^{4}C-	^{4}C-	
Nitrosospira multiformis ATCC 25196	C+	^{3}C-	^{3}C-	^{3}C-	C+	^{4}C-	^{4}C-	
Polaromonas naphthalenivorans CJ2	C-(I)	^{3}C-	^{3}C-	^{3}C-	^{4}C-	^{4}C-	^{4}C-	^{h}C+
Polaromonas sp. JS666	C-(I)	^{3}C-	^{3}C-	^{3}C-	^{4}C-	^{4}C-	^{4}C-	^{h}C+
Polynucleobacter necessarius STIR1	C-(I)	^{3}C-	^{3}C-	^{3}C-	^{4}C-	^{4}C-	^{4}C-	^{h}C+
Polynucleobacter sp. QLW-P1DMWA-1	C-(I)	^{3}C-	^{3}C-	^{3}C-	^{4}C-	^{4}C-	^{4}C-	
Ralstonia solanacearum GMI1000	C-(I)	^{3}C-	^{3}C-	^{3}C-	^{4}C-	^{4}C-	^{4}C-	^{h}C+
Rhodoferax ferrireducens T118	C-(I)	^{3}C-	^{3}C-	^{3}C-	^{4}C-	^{4}C-	^{4}C-	^{h}C+
Thiobacillus denitrificans ATCC 25259	C+	^{3}C-	^{3}C-	^{3}C-	C+	^{4}C-	^{4}C-	
Verminephrobacter eiseniae EF01-2	C-(I)	^{3}C-	^{3}C-	^{3}C-	^{4}C-	^{4}C-	^{4}C-	
**Deltaproteobacteria** (19)	**19/3**	**17/1**	**17/1**	**16/2**	**17/1**	**12/1**	**11/5**	**12/0**
Anaeromyxobacter dehalogenans 2CP-C	C+	C+	^{h}C+		C+	C+	C+	^{h}C+
Anaeromyxobacter sp. Fw109-5	C+	C+	^{h}C+	C+	C+		C+	^{h}C+
Bdellovibrio bacteriovorus HD100	C+/^{4}C-		^{4}C-		^{3}C-	^{4}C-		^{h}C+
Desulfococcus oleovorans Hxd3	C+	C+	^{h}C+	C+	C+			
Desulfotalea psychrophila LSv54	C+	C+	^{h}C+	C+	C+			^{h}C+
Desulfovibrio desulfuricans subsp. desulfuricans str. G20	C+	C+	^{h}C+	C+	C+			^{h}C+
**Desulfovibrio vulgaris**								
Desulfovibrio vulgaris subsp. vulgaris DP4	C+	C+	^{h}C+	C+	C+	C+	^{1}C-	^{h}C+
Desulfovibrio vulgaris subsp. vulgaris str. Hildenborough	C+	C+	^{h}C+	C+	C+	C+	^{1}C-	^{h}C+
Geobacter lovleyi SZ	C+	C+	^{h}C+	C+	C+	C+	C+	^{h}C+
Geobacter metallireducens GS-15	C+	C+	^{h}C+	C+	C+	C+	C+	^{h}C+

Table 1: (cont) Sequences used in the study

Taxon	S4	S14	S18	L28	L31	L32	L33	L36
Geobacter sulfurreducens PCA	C+	C+	^{h}C+	C+	C+	C+	C+	
Geobacter uraniireducens Rf4	C+	C+	^{h}C+	C+	C+	C+	C+	
Lawsonia intracellularis PHE/MN1-00	C+			C+				
Myxococcus xanthus DK 1622	C+/^{3}C-	C+/^{3}C-	^{h}C+	C+/^{3}C-	C+		C+/^{4}C-	^{h}C+
Pelobacter carbinolicus DSM 2380	C+	C+	^{h}C+	C+	C+	C+	C+	^{h}C+
Pelobacter propionicus DSM 2379	C+	C+	^{h}C+	C+	C+	C+	C+	
Sorangium cellulosum 'So ce 56'	C+/^{4}C-	C+	^{h}C+	^{4}C-	C+	C+	^{4}C-/^{1}C-	^{h}C+
Syntrophobacter fumaroxidans MPOB	C+	C+	^{h}C+	C+	C+	C+	C+	
Syntrophus aciditrophicus SB	C+	C+	^{h}C+	C+	C+	C+	C+	
**Epsilonproteobacteria** (19)	**0/19**	**16/0**	**3/16**	**0/19**	**13/6**	**0/17**	**2/12**	**11/0**
Arcobacter butzleri RM4018	C-(II)	C+	^{3}C-	^{3}C-	C+	^{4}C-	^{2}C-	^{h}C+
Campylobacter concisus 13826	C-(II)	C+	^{3}C-	^{3}C-	C+	^{4}C-		
Campylobacter curvus 525.92	C-(II)	C+	^{3}C-	^{3}C-	C+			
Campylobacter fetus subsp. fetus 82-40	C-(II)	C+	^{3}C-	^{3}C-	C+	^{4}C-		
Campylobacter hominis ATCC BAA-381	C-(II)	C+	^{3}C-	^{3}C-	C+	^{4}C-	^{1}C-	
**Campylobacter jejuni**								
Campylobacter jejuni RM1221	C-(II)	C+	^{3}C-	^{3}C-	C+	^{4}C-	^{1}C-	^{h}C+
Campylobacter jejuni subsp. doylei 269.97	C-(II)	C+	^{3}C-	^{3}C-	C+	^{4}C-	^{1}C-	
Campylobacter jejuni subsp. jejuni 81-176	C-(II)	C+	^{3}C-	^{3}C-	C+	^{4}C-	^{1}C-	^{h}C+
Campylobacter jejuni subsp. jejuni 81116	C-(II)	C+	^{3}C-	^{3}C-	C+	^{4}C-	^{1}C-	^{h}C+
Campylobacter jejuni subsp. jejuni NCTC 11168	C-(II)	C+	^{3}C-	^{3}C-	C+	^{4}C-	^{1}C-	^{h}C+
Helicobacter acinonychis str. Sheeba	C-(II)		^{3}C-	^{3}C-	^{2}C-	^{4}C-	^{1}C-	
Helicobacter hepaticus ATCC 51449	C-(II)		^{3}C-	^{3}C-	^{2}C-	^{4}C-	^{2}C-	
**Helicobacter pylori**								
Helicobacter pylori 26695	C-(II)	C+	^{3}C-	^{3}C-	^{2}C-	^{4}C-	^{1}C-	^{h}C+
Helicobacter pylori HPAG1	C-(II)	C+	^{3}C-	^{3}C-	^{2}C-	^{4}C-	^{1}C-	^{h}C+
Helicobacter pylori J99	C-(II)	C+	^{3}C-	^{3}C-	^{2}C-	^{4}C-	^{1}C-	^{h}C+
Nitratiruptor sp. SB155-2	C-(II)	C+	^{h}C+	^{3}C-	C+	^{4}C-	C+	^{h}C+
Sulfurimonas denitrificans DSM 1251	C-(II)	C+	^{h}C+	^{3}C-	C+	^{4}C-	C+	^{h}C+
Sulfurovum sp. NBC37-1	C-(II)	C+	^{h}C+	^{3}C-	C+	^{4}C-		^{h}C+
Wolinella succinogenes DSM 1740	C-(II)		^{3}C-	^{3}C-	^{2}C-			
**Gammaproteobacteria** (166)	**0/166**	**7/162**	**0/166**	**0/161**	**143/90**	**0/136**	**0/163**	**118/61**
**Acinetobacter baumannii**								
Acinetobacter baumannii ACICU	C-(I)	^{3}C-	^{3}C-	^{3}C-	C+/^{4}C-		^{4}C-	^{h}C+
Acinetobacter baumannii ATCC 17978		^{3}C-	^{3}C-	^{3}C-	C+/^{4}C-		^{4}C-	^{h}C+
Acinetobacter baumannii AYE	C-(I)	^{3}C-/^{3}C-	^{3}C-/^{3}C-	^{3}C-/^{3}C-	C+/C+/^{4}C-/^{4}C-		^{4}C-/^{4}C-	^{h}C+
Acinetobacter baumannii SDF	C-(I)	^{3}C-	^{3}C-	^{3}C-	C+/^{4}C-		^{4}C-	^{h}C+
Acinetobacter sp. ADP1	C-(I)	^{3}C-	^{3}C-	^{3}C-	C+/^{4}C-		^{4}C-	^{h}C+
**Actinobacillus pleuropneumoniae**								
Actinobacillus pleuropneumoniae L20	C-(I)	^{3}C-		^{3}C-	C+/^{4}C-		^{4}C-	^{h}C+/^{2}C-
Actinobacillus pleuropneumoniae serovar 3 str. JL03	C-(I)	^{3}C-	^{3}C-	^{3}C-	C+/^{4}C-		^{4}C-	^{h}C+/^{2}C-
Actinobacillus pleuropneumoniae serovar 7 str. AP76	C-(I)	^{3}C-	^{3}C-	^{3}C-	C+/^{4}C-		^{4}C-	^{h}C+/^{2}C-
Actinobacillus succinogenes 130Z	C-(I)	^{3}C-	^{3}C-	^{3}C-	C+		^{4}C-	^{h}C+
Aeromonas hydrophila subsp. hydrophila ATCC 7966	C-(I)	^{3}C-	^{3}C-	^{3}C-	C+/^{4}C-	^{4}C-	^{4}C-	^{2}C-
Aeromonas salmonicida subsp. salmonicida A449	C-(I)	^{3}C-	^{3}C-	^{3}C-	C+/^{4}C-	^{4}C-	^{4}C-	^{h}C+/^{2}C-
Alcanivorax borkumensis SK2	C-(I)	^{3}C-	^{3}C-	^{3}C-	C+	^{4}C-	^{4}C-	^{h}C+

Table 1: (cont) Sequences used in the study

Taxon	S4	S14	S18	L28	L31	L32	L33	L36
Vibrio fischeri ES114	C-(I)	^{3}C-	^{3}C-	^{3}C-	C+	^{4}C-	^{4}C-	^{h}C+
Alkalilimnicola ehrlichei MLHE-1	C-(I)	^{3}C-	^{3}C-	^{3}C-	^{4}C-	^{4}C-	^{4}C-	^{h}C+
**Buchnera aphidicola**								
Buchnera aphidicola str. APS (Acyrthosiphon pisum)	C-(I)	^{3}C-	^{3}C-	^{3}C-	C+	^{4}C-	^{4}C-	^{h}C+
Buchnera aphidicola str. Bp (Baizongia pistaciae)	C-(I)	^{3}C-	^{3}C-	^{3}C-	C+	^{4}C-	^{4}C-	^{h}C+
Buchnera aphidicola str. Cc (Cinara cedri)	C-(I)	^{3}C-	^{3}C-	^{3}C-	C+		^{4}C-	^{h}C+
Buchnera aphidicola str. Sg (Schizaphis graminum)	C-(I)	^{3}C-	^{3}C-	^{3}C-	C+	^{4}C-	^{4}C-	^{h}C+
Candidatus Vesicomyosocius okutanii HA	C-(I)	^{3}C-	^{3}C-	^{3}C-	C+	^{4}C-	^{4}C-	^{h}C+
Baumannia cicadellinicola str. Hc (Homalodisca coagulata)	C-(I)	^{3}C-	^{3}C-	^{3}C-	C+	^{4}C-	^{4}C-	^{h}C+
Candidatus Blochmannia floridanus	C-(I)	^{3}C-	^{3}C-	^{3}C-	C+	^{4}C-	^{4}C-	^{h}C+
Candidatus Blochmannia pennsylvanicus str. BPEN	C-(I)	^{3}C-	^{3}C-	^{3}C-	C+	^{4}C-	^{4}C-	^{h}C+
Candidatus Carsonella ruddii PV	C-(I)	^{3}C-						
Candidatus Ruthia magnifica str. Cm (Calyptogena magnifica)	C-(I)	^{3}C-	^{3}C-	^{3}C-	C+	^{4}C-	^{4}C-	^{h}C+
Chromohalobacter salexigens DSM 3043	C-(I)	^{3}C-	^{3}C-	^{3}C-	C+	^{4}C-	^{4}C-	^{h}C+
Citrobacter koseri ATCC BAA-895	C-(I)	^{3}C-	^{3}C-		^{4}C-		^{4}C-	^{2}C-
Colwellia psychrerythraea 34H	C-(I)	^{3}C-	^{3}C-	^{3}C-	C+		^{4}C-	^{h}C+
**Coxiella burnetii**								
Coxiella burnetii Dugway 5J108-111	C-(I)	^{h}C+	^{2}C-	^{3}C-	C+	^{4}C-		^{h}C+
Coxiella burnetii RSA 331	C-(I)	^{h}C+	^{2}C-	^{3}C-	C+	^{4}C-		^{h}C+
Coxiella burnetii RSA 493	C-(I)	^{h}C+	^{2}C-	^{3}C-	C+	^{4}C-		^{h}C+
Dichelobacter nodosus VCS1703A	C-(I)	^{3}C-	^{3}C-	^{3}C-	^{4}C-	^{4}C-	^{4}C-	
Enterobacter sakazakii ATCC BAA-894	C-(I)	^{3}C-	^{3}C-		C+/^{4}C-	^{4}C-	^{4}C-	^{h}C+/^{2}C-
Enterobacter sp. 638	C-(I)	^{3}C-	^{3}C-	^{3}C-	C+/^{4}C-	^{4}C-	^{4}C-	^{h}C+/^{2}C-
Erwinia tasmaniensis	C-(I)	^{3}C-	^{3}C-	^{3}C-	C+/^{4}C-	^{4}C-	^{4}C-	^{h}C+/^{2}C-
**Escherichia coli**								
Escherichia coli 536	C-(I)	^{3}C-	^{3}C-	^{3}C-	C+/^{4}C-	^{4}C-	^{4}C-	^{h}C+/^{2}C-
Escherichia coli APEC O1	C-(I)	^{3}C-			^{4}C-			
Escherichia coli ATCC 8739	C-(I)	^{3}C-	^{3}C-	^{3}C-	C+/^{4}C-	^{4}C-	^{4}C-	^{h}C+/^{2}C-
Escherichia coli CFT073	C-(I)	^{3}C-	^{3}C-	^{3}C-	^{4}C-		^{4}C-	^{2}C-
Escherichia coli E24377A	C-(I)	^{3}C-	^{3}C-	^{3}C-	C+/^{4}C-		^{4}C-	^{h}C+/^{2}C-
Escherichia coli HS	C-(I)	^{3}C-	^{3}C-	^{3}C-	C+/^{4}C-	^{4}C-	^{4}C-	^{h}C+/^{2}C-
Escherichia coli O157:H7 EDL933	C-(I)	^{3}C-	^{3}C-	^{3}C-	C+/^{4}C- /^{4}C-/^{4}C-	^{4}C-	^{4}C-	^{h}C+
Escherichia coli O157:H7 str. Sakai	C-(I)	^{3}C-	^{3}C-	^{3}C-	C+/^{4}C- /^{4}C-	^{4}C-	^{4}C-	^{h}C+/^{2}C-
Escherichia coli SMS-3-5	C-(I)	^{3}C-	^{3}C-	^{3}C-	C+/^{4}C-	^{4}C-	^{4}C-	^{h}C+/^{2}C-
Escherichia coli UTI89	C-(I)	^{3}C-	^{3}C-	^{3}C-	^{4}C-	^{4}C-	^{4}C-	^{h}C+/^{2}C-
Escherichia coli str. K12 substr. DH10B	C-(I)	^{3}C-/^{3}C-	^{3}C-/^{3}C-	^{3}C-/^{3}C-	C+/C+/^{4}C- /^{4}C-	^{4}C-/^{4}C-	^{4}C-/^{4}C-	^{h}C+/^{2}C-
Escherichia coli str. K12 substr. MG1655	C-(I)	^{3}C-/^{3}C-	^{3}C-/^{3}C-	^{3}C-/^{3}C-	C+/C+/^{4}C- /^{4}C-	^{4}C-/^{4}C-	^{4}C-/^{4}C-	^{h}C+/^{2}C-
Escherichia coli str. K12 substr. W3110	C-(I)	^{3}C-	^{3}C-	^{3}C-	C+/^{4}C-	^{4}C-	^{4}C-	^{h}C+/^{2}C-
Francisella tularensis subsp. novicida U112	C-(I)	^{2}C-	^{3}C-	^{3}C-	C+	^{4}C-	^{4}C-	^{h}C+
Francisella philomiragia subsp. philomiragia ATCC 25017	C-(I)	^{2}C-	^{3}C-	^{3}C-	C+	^{4}C-	^{4}C-	
**Francisella tularensis**								
Francisella tularensis subsp. holarctica	C-(I)	^{2}C-	^{3}C-	^{3}C-	C+	^{4}C-	^{4}C-	^{h}C+
Francisella tularensis subsp. holarctica FTNF002-00	C-(I)	^{2}C-	^{3}C-	^{3}C-	C+	^{4}C-	^{4}C-	
Francisella tularensis subsp. holarctica OSU18	C-(I)	^{2}C-	^{3}C-	^{3}C-	C+	^{4}C-	^{4}C-	
Francisella tularensis subsp. mediasiatica FSC147	C-(I)	^{2}C-	^{3}C-	^{3}C-	C+	^{4}C-	^{4}C-	^{h}C+
Francisella tularensis subsp. tularensis FSC198	C-(I)	^{2}C-	^{3}C-	^{3}C-	C+	^{4}C-	^{4}C-	^{h}C+

Table 1: (cont) Sequences used in the study

Taxon	S4	S14	S18	L28	L31	L32	L33	L36
Francisella tularensis subsp. tularensis SCHU S4	C-(I)	^{2}C-	^{3}C-	^{3}C-	C+	^{4}C-	^{4}C-	^{h}C+
Francisella tularensis subsp. tularensis WY96-3418	C-(I)	^{2}C-	^{3}C-	^{3}C-	C+	^{4}C-	^{4}C-	^{h}C+
Haemophilus ducreyi 35000HP	C-(I)	^{3}C-	^{3}C-	^{3}C-	C+/^{4}C-		^{4}C-	^{h}C+/^{2}C-
**Haemophilus influenzae**								
Haemophilus influenzae 86-028NP	C-(I)	^{3}C-	^{3}C-	^{3}C-	C+		^{4}C-	
Haemophilus influenzae PittEE	C-(I)	^{3}C-	^{3}C-	^{3}C-	C+		^{4}C-	^{h}C+
Haemophilus influenzae PittGG	C-(I)	^{3}C-	^{3}C-	^{3}C-	C+		^{4}C-	
Haemophilus influenzae Rd KW20	C-(I)	^{3}C-	^{3}C-	^{3}C-	C+		^{4}C-	^{h}C+
Hahella chejuensis KCTC 2396	C-(I)	^{3}C-	^{3}C-	^{3}C-	C+		^{4}C-	^{h}C+
Halorhodospira halophila SL1	C-(I)	^{3}C-	^{3}C-	^{3}C-	^{4}C-	^{4}C-	^{4}C-	^{h}C+
**Histophilus somni**								
Haemophilus somnus 129PT	C-(I)	^{3}C-	^{3}C-	^{3}C-	C+		^{4}C-	^{h}C+
Haemophilus somnus 2336	C-(I)	^{3}C-	^{3}C-	^{3}C-	C+		^{4}C-	^{h}C+
Idiomarina loihiensis L2TR	C-(I)	^{3}C-	^{3}C-	^{3}C-	C+		^{4}C-	^{h}C+
Klebsiella pneumoniae subsp. pneumoniae MGH 78578	C-(I)	^{3}C-	^{3}C-	^{3}C-	C+/^{4}C-	^{4}C-	^{4}C-	^{2}C-
**Legionella pneumophila**								
Legionella pneumophila str. Corby	C-(I)	C+	^{3}C-	^{3}C-	C+	^{4}C-	^{4}C-	
Legionella pneumophila str. Lens	C-(I)	C+	^{3}C-	^{3}C-	C+	^{4}C-	^{4}C-	^{h}C+
Legionella pneumophila str. Paris	C-(I)	C+	^{3}C-	^{3}C-	C+	^{4}C-	^{4}C-	^{h}C+
Legionella pneumophila subsp. pneumophila str. Philadelphia 1	C-(I)	C+	^{3}C-	^{3}C-	C+	^{4}C-	^{4}C-	^{h}C+
Mannheimia succiniciproducens MBEL55E	C-(I)	^{3}C-	^{3}C-	^{3}C-	C+		^{4}C-	^{h}C+
Marinobacter aquaeolei VT8	C-(I)	^{3}C-	^{3}C-	^{3}C-	C+	^{4}C-	^{4}C-	^{h}C+
Marinomonas sp. MWYL1	C-(I)	^{3}C-	^{3}C-	^{3}C-	C+	^{4}C-	^{4}C-	^{h}C+
Methylococcus capsulatus str. Bath	C-(I)	^{3}C-	^{3}C-	^{3}C-	C+	^{4}C-	^{4}C-	^{h}C+
Nitrosococcus oceani ATCC 19707	C-(I)	^{3}C-	^{3}C-	^{3}C-	C+	^{4}C-	^{4}C-	^{h}C+
Pasteurella multocida subsp. multocida str. Pm70	C-(I)	^{3}C-	^{3}C-	^{3}C-	C+/^{4}C-		^{4}C-	^{h}C+
Pectobacterium atrosepticum SCRI1043	C-(I)	^{3}C-	^{3}C-	^{3}C-	C+/^{4}C-	^{4}C-	^{4}C-	^{h}C+
Photobacterium profundum SS9	C-(I)	^{3}C-	^{3}C-	^{3}C-	C+	^{4}C-	^{4}C-	^{h}C+
Photorhabdus luminescens subsp. laumondii TTO1	C-(I)	^{3}C-	^{3}C-	^{3}C-	C+/^{4}C-	^{4}C-	^{4}C-	^{2}C-
Pseudoalteromonas atlantica T6c	C-(I)	^{3}C-	^{3}C-	^{3}C-	C+	^{4}C-	^{4}C-	^{h}C+
Pseudoalteromonas haloplanktis TAC125	C-(I)	^{3}C-	^{3}C-	^{3}C-	C+	^{4}C-	^{4}C-	^{2}C-
**Pseudomonas aeruginosa**								
Pseudomonas aeruginosa PA7	C-(I)	^{3}C-	^{3}C-	^{3}C-	C+/^{4}C-	^{4}C-	^{4}C-	^{h}C+/^{2}C-
Pseudomonas aeruginosa PAO1	C-(I)	^{3}C-	^{3}C-	^{3}C-	C+/^{4}C-	^{4}C-	^{4}C-	^{h}C+/^{2}C-
Pseudomonas aeruginosa UCBPP-PA14	C-(I)	^{3}C-	^{3}C-	^{3}C-	C+/^{4}C-	^{4}C-	^{4}C-	^{h}C+/^{2}C-
Pseudomonas entomophila L48	C-(I)	^{3}C-	^{3}C-	^{3}C-	C+/^{4}C-	^{4}C-	^{4}C-	^{h}C+
**Pseudomonas fluorescens**								
Pseudomonas fluorescens Pf-5	C-(I)	^{3}C-	^{3}C-	^{3}C-	C+/^{4}C-	^{4}C-	^{4}C-	^{2}C-
Pseudomonas fluorescens PfO-1	C-(I)	^{3}C-	^{3}C-	^{3}C-	C+	^{4}C-	^{4}C-	
Pseudomonas mendocina ymp	C-(I)	^{3}C-	^{3}C-	^{3}C-	C+	^{4}C-	^{4}C-	^{h}C+
**Pseudomonas putida**								
Pseudomonas putida F1	C-(I)	^{3}C-	^{3}C-	^{3}C-	C+	^{4}C-	^{4}C-	^{h}C+
Pseudomonas putida GB-1	C-(I)	^{3}C-	^{3}C-	^{3}C-	C+	^{4}C-	^{4}C-	
Pseudomonas putida KT2440	C-(I)	^{3}C-	^{3}C-	^{3}C-	C+	^{4}C-	^{4}C-	^{h}C+
Pseudomonas putida W619	C-(I)	^{3}C-	^{3}C-	^{3}C-	C+	^{4}C-	^{4}C-	^{h}C+
Pseudomonas syringae pv. phaseolicola 1448A	C-(I)	^{3}C-	^{3}C-	^{3}C-	C+	^{4}C-	^{4}C-	^{h}C+

Table 1: (cont) Sequences used in the study

Taxon	S4	S14	S18	L28	L31	L32	L33	L36
Pseudomonas stutzeri A1501	C-(I)	^{3}C-	^{3}C-	^{3}C-	^{4}C-	^{4}C-	^{4}C-	^{h}C+
Pseudomonas syringae pv. syringae B728a	C-(I)	^{3}C-	^{3}C-	^{3}C-	C+/^{4}C-	^{4}C-	^{4}C-	
Pseudomonas syringae pv. tomato str. DC3000	C-(I)	^{3}C-	^{3}C-	^{3}C-	C+/^{4}C-	^{4}C-	^{4}C-	^{h}C+
Psychrobacter arcticus 273-4	C-(I)	^{3}C-	^{3}C-	^{3}C-	^{4}C-		^{4}C-	^{h}C+
Psychrobacter cryohalolentis K5	C-(I)	^{3}C-	^{3}C-	^{3}C-	^{4}C-		^{4}C-	^{h}C+
Psychrobacter sp. PRwf-1	C-(I)	^{3}C-	^{3}C-	^{3}C-	^{4}C-		^{4}C-	
Psychromonas ingrahamii 37	C-(I)/C-(I)	^{3}C-	^{2}C-	^{3}C-	C+	^{4}C-	^{4}C-	^{h}C+/^{h}C+
Saccharophagus degradans 2-40	C-(I)	^{2}C-	^{3}C-	^{3}C-	C+	^{4}C-	^{4}C-	^{h}C+
**Salmonella enterica**								
Salmonella enterica subsp. arizonae serovar 62:z4,z23:–	C-(I)	^{3}C-	^{3}C-		C+/^{4}C-	^{4}C-	^{4}C-	
Salmonella enterica subsp. enterica serovar Choleraesuis str. SC-B67	C-(I)	^{3}C-	^{3}C-	^{3}C-	C+/^{4}C-	^{4}C-	^{4}C-	^{h}C+/^{2}C-
Salmonella enterica subsp. enterica serovar Paratyphi B str. SPB7	C-(I)	^{3}C-	^{3}C-	^{4}C-	C+/^{4}C-		^{4}C-	^{2}C-
Salmonella enterica subsp. enterica serovar Typhi str. CT18	C-(I)	^{3}C-	^{3}C-	^{3}C-	C+/^{4}C-	^{4}C-	^{4}C-	^{h}C+/^{2}C-
Salmonella enterica subsp. enterica serovar Typhi str. Ty2	C-(I)	^{3}C-	^{3}C-	^{3}C-	C+/^{4}C-	^{4}C-	^{4}C-	^{h}C+/^{2}C-
Salmonella enterica subsp. enterica serovar Paratyphi A str. ATCC 9150	C-(I)	^{3}C-	^{3}C-	^{3}C-	C+/^{4}C-	^{4}C-	^{4}C-	^{h}C+/^{2}C-
Salmonella typhimurium LT2	C-(I)	^{3}C-	^{3}C-	^{3}C-	C+/^{4}C-	^{4}C-	^{4}C-	^{h}C+/^{2}C-
Serratia proteamaculans 568	C-(I)	^{3}C-	^{3}C-	^{3}C-	C+/^{4}C-	^{4}C-	^{4}C-	^{2}C-
Shewanella amazonensis SB2B	C-(I)	^{3}C-	^{3}C-	^{3}C-	C+	^{4}C-	^{4}C-	^{h}C+
**Shewanella baltica**								
Shewanella baltica OS155	C-(I)	^{3}C-	^{3}C-	^{3}C-	C+	^{4}C-	^{4}C-	^{h}C+
Shewanella baltica OS185	C-(I)	^{3}C-	^{3}C-	^{3}C-	C+	^{4}C-	^{4}C-	^{h}C+
Shewanella baltica OS195	C-(I)	^{3}C-	^{3}C-	^{3}C-	C+	^{4}C-	^{4}C-	
Shewanella denitrificans OS217	C-(I)	^{3}C-	^{3}C-	^{3}C-	C+	^{4}C-	^{4}C-	^{h}C+
Shewanella frigidimarina NCIMB 400	C-(I)	^{3}C-	^{3}C-	^{3}C-	C+	^{4}C-	^{4}C-	^{h}C+
Shewanella halifaxensis HAW-EB4	C-(I)	^{3}C-	^{3}C-	^{3}C-	C+	^{4}C-	^{4}C-	
Shewanella loihica PV-4	C-(I)	^{3}C-	^{3}C-	^{3}C-	C+	^{4}C-	^{4}C-	^{h}C+
Shewanella oneidensis MR-1	C-(I)	^{3}C-	^{3}C-	^{3}C-	C+	^{4}C-	^{4}C-	^{h}C+
Shewanella pealeana ATCC 700345	C-(I)	^{3}C-	^{3}C-	^{3}C-	C+	^{4}C-	^{4}C-	
Shewanella putrefaciens CN-32	C-(I)	^{3}C-	^{3}C-	^{3}C-	C+	^{4}C-	^{4}C-	^{h}C+
Shewanella sediminis HAW-EB3	C-(I)	^{3}C-	^{3}C-	^{3}C-	C+	^{4}C-	^{4}C-	
Shewanella sp. ANA-3	C-(I)	^{3}C-	^{3}C-	^{3}C-	C+	^{4}C-	^{4}C-	^{h}C+
Shewanella sp. MR-4	C-(I)	^{3}C-	^{3}C-	^{3}C-	C+	^{4}C-	^{4}C-	^{h}C+
Shewanella sp. MR-7	C-(I)	^{3}C-	^{3}C-	^{3}C-	C+	^{4}C-	^{4}C-	^{h}C+
Shewanella sp. W3-18-1	C-(I)	^{3}C-	^{3}C-	^{3}C-	C+	^{4}C-	^{4}C-	^{h}C+
Shewanella woodyi ATCC 51908	C-(I)	^{3}C-	^{3}C-	^{3}C-	C+	^{4}C-	^{4}C-	^{h}C+
**Shigella boydii**								
Shigella boydii CDC 3083-94	C-(I)	^{3}C-	^{3}C-	^{3}C-	C+	^{4}C-	^{4}C-	
Shigella boydii Sb227	C-(I)	^{3}C-	^{3}C-	^{3}C-	C+/^{4}C-	^{4}C-	^{4}C-	^{h}C+
Shigella dysenteriae Sd197	C-(I)	^{3}C-	^{3}C-	^{3}C-	C+	^{4}C-	^{4}C-	^{h}C+
**Shigella flexneri**								
Shigella flexneri 2a str. 2457T	C-(I)	^{3}C-	^{3}C-	^{3}C-	C+	^{4}C-	^{4}C-	^{h}C+
Shigella flexneri 2a str. 301	C-(I)	^{3}C-	^{3}C-	^{3}C-	C+	^{4}C-	^{4}C-	^{h}C+
Shigella flexneri 5 str. 8401	C-(I)	^{3}C-	^{3}C-				^{4}C-	^{h}C+
Shigella sonnei Ss046	C-(I)	^{3}C-	^{3}C-	^{3}C-	C+	^{4}C-	^{4}C-	^{h}C+
Sodalis glossinidius str. 'morsitans'	C-(I)	^{3}C-	^{3}C-	^{3}C-	C+	^{4}C-	^{4}C-	^{h}C+
Stenotrophomonas maltophilia K279a	C-(I)	^{3}C-	^{3}C-	^{3}C-	^{4}C-	^{4}C-	^{4}C-	^{2}C-

Table 1: (cont) Sequences used in the study

Taxon	S4	S14	S18	L28	L31	L32	L33	L36
Thiomicrospira crunogena XCL-2	C-(I)	^{3}C-	^{3}C-	^{3}C-	^{4}C-	^{4}C-	^{4}C-	^{3}C-
**Vibrio cholerae**								
Vibrio cholerae O1 biovar El Tor str. N16961	C-(I)	^{3}C-	^{3}C-	^{3}C-	C+/^{4}C-	^{4}C-	^{4}C-	^{h}C+/^{2}C-
Vibrio cholerae O395	C-(I)	^{3}C-	^{3}C-	^{3}C-	C+/^{4}C-	^{4}C-	^{4}C-	^{h}C+/^{2}C-
Vibrio harveyi ATCC BAA-1116	C-(I)	^{3}C-	^{3}C-	^{3}C-	C+/^{4}C-	^{4}C-	^{4}C-	^{h}C+/^{2}C-
Vibrio parahaemolyticus RIMD 2210633	C-(I)	^{3}C-	^{3}C-	^{3}C-	C+/^{4}C-	^{4}C-	^{4}C-	^{h}C+
**Vibrio vulnificus**								
Vibrio vulnificus CMCP6	C-(I)	^{3}C-	^{3}C-	^{3}C-	C+/^{4}C-	^{4}C-	^{4}C-	
Vibrio vulnificus YJ016	C-(I)	^{3}C-	^{3}C-	^{3}C-	C+/^{4}C-	^{4}C-	^{4}C-	^{2}C-
Wigglesworthia glossinidia endosymbiont of Glossina brevipalpis	C-(I)	^{3}C-	^{3}C-	^{3}C-	C+	^{4}C-		^{h}C+
Xanthomonas axonopodis pv. citri str. 306	C-(I)	^{3}C-	^{3}C-	^{3}C-	^{4}C-	^{4}C-	^{4}C-	^{2}C-
**Xanthomonas campestris**								
Xanthomonas campestris pv. campestris	C-(I)	^{3}C-	^{3}C-	^{3}C-	^{4}C-	^{4}C-	^{4}C-	^{2}C-
Xanthomonas campestris pv. campestris str. 8004	C-(I)	^{3}C-	^{3}C-	^{3}C-	^{4}C-	^{4}C-	^{4}C-	^{2}C-
Xanthomonas campestris pv. campestris str. ATCC 33913	C-(I)	^{3}C-	^{3}C-	^{3}C-	^{4}C-	^{4}C-	^{4}C-	^{2}C-
Xanthomonas campestris pv. vesicatoria str. 85-10	C-(I)	^{3}C-	^{3}C-	^{3}C-	^{4}C-	^{4}C-	^{4}C-	^{2}C-
**Xanthomonas oryzae**								
Xanthomonas oryzae pv. oryzae KACC10331	C-(I)	^{3}C-	^{3}C-		^{4}C-	^{4}C-	^{4}C-	
Xanthomonas oryzae pv. oryzae MAFF 311018	C-(I)	^{3}C-	^{3}C-	^{3}C-	^{4}C-	^{4}C-	^{4}C-	^{2}C-
**Xylella fastidiosa**								
Xylella fastidiosa 9a5c	C-(I)	^{3}C-	^{3}C-	^{3}C-	^{4}C-	^{4}C-	^{4}C-	^{2}C-
Xylella fastidiosa M12	C-(I)	^{3}C-	^{3}C-	^{3}C-	^{4}C-	^{4}C-	^{4}C-	^{2}C-
Xylella fastidiosa M23	C-(I)	^{3}C-	^{3}C-	^{3}C-	^{4}C-	^{4}C-	^{4}C-	^{2}C-
Xylella fastidiosa Temecula1	C-(I)	^{3}C-	^{3}C-	^{3}C-	^{4}C-	^{4}C-	^{4}C-	^{2}C-
Yersinia enterocolitica subsp. enterocolitica 8081	C-(I)	^{3}C-	^{3}C-	^{3}C-	C+/^{4}C-	^{4}C-	^{4}C-	^{h}C+/^{2}C-
**Yersinia pestis**								
Yersinia pestis Angola	C-(I)	^{3}C-	^{3}C-	^{3}C-	C+	^{4}C-	^{4}C-	^{2}C-
Yersinia pestis Antiqua	C-(I)	^{3}C-	^{3}C-	^{3}C-	C+/^{4}C-	^{4}C-	^{4}C-	^{2}C-
Yersinia pestis CO92	C-(I)	^{3}C-	^{3}C-	^{3}C-	C+/^{4}C-	^{4}C-	^{4}C-	^{h}C+/^{2}C-
Yersinia pestis KIM	C-(I)	^{3}C-	^{3}C-		C+/^{4}C-	^{4}C-	^{4}C-	
Yersinia pestis Nepal516	C-(I)	^{3}C-	^{3}C-	^{3}C-	C+/^{4}C-	^{4}C-	^{4}C-	^{2}C-
Yersinia pestis Pestoides F	C-(I)	^{3}C-	^{3}C-	^{3}C-	C+/^{4}C-	^{4}C-	^{4}C-	^{2}C-
Yersinia pestis biovar Microtus str. 91001	C-(I)	^{3}C-	^{3}C-	^{3}C-	C+/^{4}C-	^{4}C-	^{4}C-	^{h}C+/^{2}C-
**Yersinia pseudotuberculosis**								
Yersinia pseudotuberculosis IP 31758	C-(I)	^{3}C-	^{3}C-	^{3}C-	C+/^{4}C-	^{4}C-	^{4}C-	^{2}C-
Yersinia pseudotuberculosis IP 32953	C-(I)	^{3}C-	^{3}C-	^{3}C-	C+/^{4}C-	^{4}C-	^{4}C-	^{h}C+/^{2}C-
Yersinia pseudotuberculosis YPIII	C-(I)	^{3}C-	^{3}C-	^{3}C-	C+/^{4}C-	^{4}C-	^{4}C-	^{h}C+/^{2}C-
**Unknown (class)** (1)	**1/0**	**1/0**	**1/0**	**0/1**	**1/0**	**1/0**	**1/0**	**1/0**
Magnetococcus sp. MC-1	C+	C+	^{h}C+	^{4}C-	C+	C+	C+	^{h}C+
**Spirochaetes** (12)	**0/13**	**12/0**	**5/7**	**3/9**	**9/3**	**7/0**	**0/6**	**11/0**
**Spirochaetes (class)** (12)	**0/13**	**12/0**	**5/7**	**3/9**	**9/3**	**7/0**	**0/6**	**11/0**
Borrelia afzelii PKo	^{4}C-	C+	^{2}C-	^{3}C-	^{4}C-		^{2}C-	^{h}C+
Borrelia burgdorferi B31	^{4}C-	C+	^{2}C-	^{3}C-	^{4}C-		^{2}C-	^{h}C+
Borrelia garinii PBi	^{4}C-	C+	^{2}C-	^{3}C-	^{4}C-		^{2}C-	^{h}C+
**Leptospira biflexa**								
Leptospira biflexa serovar Patoc strain ’Patoc 1 (Ames)’	C-(III)	C+	^{h}C+	^{3}C-	C+			^{h}C+

Table 1: (cont) Sequences used in the study

Taxon	S4	S14	S18	L28	L31	L32	L33	L36
Leptospira biflexa serovar Patoc strain 'Patoc 1 (Paris)'	C-(III)		^{h}C+	^{3}C-	C+			
**Leptospira borgpetersenii**								
Leptospira borgpetersenii serovar Hardjo-bovis JB197	C-(III)	C+	^{3}C-	^{3}C-	C+	C+		^{h}C+
Leptospira borgpetersenii serovar Hardjo-bovis L550	C-(III)/C-(III)	C+/C+	^{3}C-	^{3}C-	C+	C+		^{h}C+/^{h}C+
**Leptospira interrogans**								
Leptospira interrogans serovar Copenhageni str. Fiocruz L1-130	C-(III)	C+	^{3}C-	^{3}C-	C+	C+		^{h}C+
Leptospira interrogans serovar Lai str. 56601	C-(III)	C+	^{3}C-	^{3}C-	C+	C+		
Treponema denticola ATCC 35405	^{4}C-	C+	^{h}C+	C+	C+	C+	^{1}C-	^{h}C+
**Treponema pallidum**								
Treponema pallidum subsp. pallidum SS14	^{4}C-	C+	^{h}C+	C+	C+	C+	^{1}C-	^{h}C+
Treponema pallidum subsp. pallidum str. Nichols	^{4}C-	C+	^{h}C+	C+	C+	C+	^{1}C-	^{h}C+
**Tenericutes** (19)	**0/18**	**15/3**	**10/9**	**0/19**	**18/1**	**15/3**	**17/1**	**12/3**
**Mollicutes** (19)	**0/18**	**15/3**	**10/9**	**0/19**	**18/1**	**15/3**	**17/1**	**12/3**
Acholeplasma laidlawii PG-8A	^{4}C-	^{4}C-	^{3}C-	^{3}C-	^{4}C-	^{4}C-	C+/^{1}C-	^{4}C-
Aster yellows witches'-broom phytoplasma AYWB	^{4}C-	^{4}C-	^{3}C-	^{3}C-	C+	^{3}C-	^{s}C+	^{4}C-
Mesoplasma florum L1	^{4}C-	C+	^{4}C-	^{4}C-	C+	C+	C+	^{h}C+
Mycoplasma agalactiae PG2	^{4}C-	C+	^{h}C+	^{4}C-	C+	C+	C+	
Mycoplasma capricolum subsp. capricolum ATCC 27343	^{4}C-	C+	^{4}C-	^{4}C-	C+	C+	C+	^{h}C+
Mycoplasma gallisepticum R	^{4}C-	C+	^{h}C+	^{4}C-	C+	C+	C+	^{h}C+
Mycoplasma genitalium G37	^{4}C-	C+	^{h}C+	^{4}C-	C+	C+	C+	^{h}C+
**Mycoplasma hyopneumoniae**								
Mycoplasma hyopneumoniae 232	^{4}C-	C+	^{h}C+	^{4}C-	C+			
Mycoplasma hyopneumoniae 7448	^{4}C-	C+	^{h}C+	^{4}C-	C+	C+		^{h}C+
Mycoplasma hyopneumoniae J	^{4}C-	C+	^{h}C+	^{4}C-	C+	C+		^{h}C+
Mycoplasma mobile 163K	^{4}C-	C+	^{2h}C+	^{4}C-	C+	C+	C+	^{h}C+
Mycoplasma mycoides subsp. mycoides SC str. PG1	^{4}C-		^{4}C-	^{4}C-	C+	C+		
Mycoplasma penetrans HF-2	^{4}C-	C+	^{h}C+	^{4}C-	C+	C+	C+	
Mycoplasma pneumoniae M129	^{4}C-	C+	^{h}C+	^{4}C-	C+	C+	C+/C+	^{h}C+
Mycoplasma pulmonis UAB CTIP	^{4}C-	C+	^{4}C-	^{4}C-	C+	C+		^{h}C+
Mycoplasma synoviae 53	^{4}C-	C+	^{h}C+	^{4}C-	C+	C+	C+	^{h}C+
Onion yellows phytoplasma OY-M		^{4}C-	^{3}C-	^{3}C-	C+	^{3}C-	^{s}C+/C+	^{4}C-
**Ureaplasma parvum**								
Ureaplasma parvum serovar 3 str. ATCC 27815	^{4}C-	C+	^{3}C-	^{4}C-	C+	C+	^{s}C+	^{h}C+
Ureaplasma parvum serovar 3 str. ATCC 700970	^{4}C-	C+	^{3}C-	^{4}C-	C+	C+	^{s}C+/C+	^{h}C+
**Thermotogae** (7)	**6/1**	**7/0**	**7/0**	**7/0**	**7/0**	**7/0**	**7/0**	**7/0**
**Thermotogae (class)** (7)	**6/1**	**7/0**	**7/0**	**7/0**	**7/0**	**7/0**	**7/0**	**7/0**
Fervidobacterium nodosum Rt17-B1	C+	C+	^{h}C+	C+	C+	C+	C+	^{h}C+
Petrotoga mobilis SJ95	C-(I)	C+	^{h}C+	C+	C+	C+	C+	^{h}C+
Thermosipho melanesiensis BI429	C+	C+	^{h}C+	C+	C+	C+	C+	^{h}C+
Thermotoga lettingae TMO	C+	C+	^{h}C+	C+	C+	C+	C+	^{h}C+
Thermotoga maritima MSB8	C+	C+	^{h}C+	C+	C+	C+	C+	^{h}C+
Thermotoga petrophila RKU-1	C+	C+	^{h}C+	C+	C+	C+	C+	^{h}C+
Thermotoga sp. RQ2	C+	C+	^{h}C+	C+	C+	C+	C+	^{h}C+
**Verrucomicrobia** (7)	**0/7**	**0/1**	**0/1**	**0/1**	**0/1**	**0/1**	**0/1**	**0/1**
**Opitutae** (2)	**0/2**	**0/1**	**0/1**	**0/1**	**0/1**	**0/1**	**0/1**	**0/1**
Opitutaceae bacterium TAV2 *	^{4}C-							

Table 1: (cont) Sequences used in the study

Taxon	S4	S14	S18	L28	L31	L32	L33	L36
Opitutus terrae PB90-1	^{4}C-	^{3}C-	^{4}C-	^{3}C-	^{4}C-	^{4}C-	^{3}C-	^{3}C-
**Spartobacteria** (1)	**0/1**	**0/0**	**0/0**	**0/0**	**0/0**	**0/0**	**0/0**	**0/0**
Chthoniobacter flavus Ellin428 *	^{4}C-							
**Unknown (class)** (1)	**0/1**	**0/0**	**0/0**	**0/0**	**0/0**	**0/0**	**0/0**	**0/0**
Methylacidiphilum infernorum V4 *	^{4}C-							
**Verrucomicrobiae** (3)	**0/3**	**0/0**	**0/0**	**0/0**	**0/0**	**0/0**	**0/0**	**0/0**
Akkermansia muciniphila ATCC BAA-835 *	^{4}C-							
Verrucomicrobium spinosum DSM 4136 *	^{4}C-							
Bacterium Ellin514 *	^{4}C-							

a) numbers in the parenthesis indicate the number of genomes in this level of taxonomy; b) pairs of numbers in bold indicate number of C+/C- genes detected, including all the paralogs; c) ^{h}C+ indicates a zinc binding motif with three cysteines and one histidine, similar for ^{s}C+; d) ^{4}C- indicates that the r-protein detected is missing four cysteines, ^{3}C- indicates that the r-protein is missing three cysteines, similar for ^{2}C- and ^{1}C-; e) organisms with an (*) asterisk after their names are draft genomes, which are only used in the S4 analysis.
